# Sustainable aromatic polyesters with 1,5-disubstituted indole units†

**DOI:** 10.1039/d1ra02197d

**Published:** 2021-05-05

**Authors:** Ping Wang, Baozhong Zhang

**Affiliations:** Centre of Analysis and Synthesis, Lund University P.O. Box 124 SE-22100 Lund Sweden baozhong.zhang@chem.lu.se

## Abstract

This work aims to unravel the impact of disubstitution patterns on the physical properties and processing characteristics of indole-based aromatic polyesters. A series of hydroxyl-carboxylate (AB-type) monomers with 1,5-disubstituted indole and 3–6 methylene units was conveniently synthesized and used in bulk polycondensation to yield the corresponding polyesters with decent molecular weight. These new monomers and polyesters showed enhanced thermal stability compared to the previously reported monomers and polyesters with a 1,3-disubstituted indole structure. According to DSC results, these polyesters showed tunable glass transition temperatures (*T*_g_ ∼57–80 °C), depending on the length of the aliphatic methylene units. DSC and WAXD measurements revealed that these polymers did not crystalize from melt, but the ones with 3 or 5 methylene units per repeating unit crystalized from solution. Finally, we demonstrated that the new polyesters with 1,5-disubstituted indole units could be crosslinked using sustainable aromatic aldehyde, which could further enhance their thermal properties.

## Abstract 2

1,5-disubstituted indole was investigated as new sustainable aromatic units for polyesters to enhance thermal stability.

## Graphical abstract

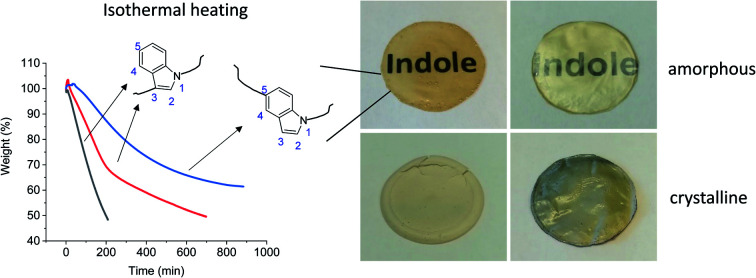

## 1. Introduction

PET is a widely used polyester for many applications (*e.g.* textiles, packaging, thermoforming for manufacturing, engineering resins), thanks to its desirable thermal and mechanical properties.^1–5^ These desirable properties are largely endowed by its rigid aromatic units (*i.e.* terephthalates) in the backbone, without which the aliphatic polyesters (*e.g.* polylactide, polycaprolactone, polyhydroxyalkanoates, *etc.*) usually exhibit inferior thermal and mechanical properties.^6–8^ Another aromatic unit naphthalene (*i.e.* naphthalate) that is larger than benzene has also been used for a commercial polyester, polyethylene naphthalate (PEN), which has enhanced thermal (*T*_g_ ∼ 120 °C), mechanical, and barrier properties compared to PET.^9,10^ Unfortunately, both terephthalate and naphthalate are produced in industry from fossil resources, which are non-sustainable.^11–15^ Bio-based terephthalate has been produced in laboratory scale,^16–18^ but its industrial production remains challenging due to the synthetic complexity and high costs. As such, alternative aromatic units from renewable resources have received growing attention.^19–21^

New sustainable aromatic building blocks for polyester synthesis have been frequently reported using various biomass resources (*e.g.* sugar, lignin, cinnamic acid, *etc.*).^22–31^ Among these building blocks, furan-based ones have attracted the most attention.^32–39^ Particularly, 2,5-furan-dicarboxylic acid (FDCA) has been conveniently produced from sugar resources and used to synthesize various polyesters.^40–46^ For example, polyethylene furanoate (PEF) is a fully biobased polyester, which can be prepared using FDCA and bio-based ethylene glycol. PEF shows favorable thermal and mechanical properties and superior barrier properties than PET,^47,48^ so it is expected to become the sustainable plastic bottle materials for next generation.^49^ Enlightened by the rapid development of PEF, we have recently initiated investigation on the use of another sustainable aromatic unit, indole, for polyester backbone structures.^50^

Indole is a large aromatic unit that widely exists in nature and wastewater streams, animal feces, and is frequently used in industry.^51–53^ There are several bio-based production routes for indole, such as thermal conversion and ammonization of furfural,^54^ pyrolysis of micro-algae,^55^ microbial synthesis from glucose,^56^ and direct conversion from bio-based aniline and ethylene glycol.^57^ In 2018, we reported our first indole-based dicarboxylate monomer with aromatic and aliphatic ester groups, which was used to produce a series of polyesters with high glass transition temperatures (*T*_g_ 55–99 °C) and low coloration.^50^ To further enhance the thermal stability, indole-based AB and AABB monomers with only aromatic ester groups were developed afterward, which were used to fabricate new indole-based polyesters with enhanced thermal stability and *T*_g_ (up to 113 °C).^58,59^ It should be noted that all our previously reported indole-based polyesters contain the same di-substitution pattern (*i.e.* 1,3-disubstituted indole units). Since di-substitution patterns of benzene or furan units have shown significant impact on polyester properties,^60–63^ it is expected that different di-substitution patterns of indole will also exert significant impact on the thermal and processing characteristics of polyesters. This structure feature can be utilized to optimize the molecular design when using indole as a new sustainable aromatic unit for polyesters.

Herein, we report on facile synthesis and polymerization of a series of 1,5-disubstituted indole-based monomers with a carboxylate ester and a hydroxyl group (AB monomers) and 3–6 methylene units. The molecular structures, thermal properties, and processability of the resulting polyesters were characterized and compared to the previously reported AB-type polyesters with isomeric 1,3-disubstituted indole units. As a result, the new polyesters with 1,5-disubstituted indole units showed further enhanced thermal stability. Finally, crosslinking of these new polyesters using a sustainable aldehyde was also investigated.

## 2. Experimental section

### 2.1. Chemicals and materials

Methyl indole-5-carboxylate (>98%), 6-bromo-1-hexanol (>97%), dibutyltin oxide (DBTO) (>98%), potassium carbonate (K_2_CO_3_) and 3-bromo-1-propanol (>97%) were purchased from Sigma-Aldrich. 5-Bromo-1-pentanol (>90%) and 4-bromo-1-butanol (technical, >80%) were purchased from TCI. Dimethylformamide (DMF) (ACS, Reag. Ph. Eur.), diethyl ether (ACS, Reag. Ph. Eur.), chloroform (analytical grade, stabilized with ethanol), magnesium sulfate (MgSO_4_), tetrahydrofuran (THF) (ACS, Reag. Ph. Eur.), hexafluoroisopropanol (HFIP) (ACS, Reag. Ph. Eur.), and ethyl acetate (EtOAc) (ACS, Reag. Ph. Eur.) were purchased from VWR Chemicals. *Tert*-butyl-(4-chlorobutoxy)dimethylsilane (98%), tetrabutylammonium fluoride hydrate (TBAF, 98%), NaH (60% dispersion in mineral oil), iodine (≥99.8%) and 3,4-dimethoxybenzaldehyde (99%) were purchased from Sigma-Aldrich. All chemicals and reagents were used as received without purification.

### 2.2. Monomer synthesis

#### 2.2.1. Monomer 3a

To a well-stirred solution of methyl indole-5-carboxylate (1, 5.00 g, 28.5 mmol) in DMF (100 mL) was added K_2_CO_3_ (15.8 g, 4.00 eq.) and 3-bromo-1-propanol (2a, 4.73 g, 1.20 eq.) dropwise. The reaction mixture was stirred overnight at room temperature. Afterward, the crude reaction mixture was extracted with EtOAc (50 mL), washed with water, dried over MgSO_4_, and concentrated *in vacuo*. The residue was purified by column chromatography (SiO_2_, EtOAc/diethyl ether 1 : 3) to yield 3a as a light-yellow oil (4.59 g, 69%). ^1^H NMR (400.13 MHz, CDCl_3_): *δ* 8.39 (dd, 1H, *J* = 1.6, 0.8 Hz); 7.91 (dd, 1H, *J* = 8.8, 1.6 Hz); 7.39 (d, 1H, *J* = 8.8 Hz); 7.19 (d, 1H, *J* = 3.2 Hz); 6.60 (dd, 1H, *J* = 3.2, 0.8 Hz); 4.32 (t, 2H, *J* = 6.8 Hz); 3.93 (s, 3H), 3.62 (t, 2H, *J* = 6.0 Hz); 2.11–2.04 (m, 2H). ^13^C NMR (100.61 MHz, CDCl_3_): *δ* 168.4, 138.7, 129.5, 128.2, 124.2, 123.0, 121.5, 109.1, 103.0, 59.4, 52.0, 43.0, 32.8. FT-IR (cm^−1^) *ν*_max_: 3417 (OH), 2930 (CH), 1705 (C=O). HRMS (ESI+) calcd for C_13_H_15_NO_3_, 234.1130, found 234.1131.

#### 2.2.2. Monomer 3b

To a well-stirred solution of methyl indole-5-carboxylate (1, 5.00 g, 28.5 mmol) in DMF (100 mL) was added a suspension of NaH (60% dispersion in mineral oil, 1.14 g, 28.5 mmol) and *tert*-butyl-(4-chlorobutoxy)dimethylsilane dropwise (0.732 mL, 28.5 mmol). The reaction mixture was stirred overnight at room temperature. Afterward, the crude reaction mixture was extracted with EtOAc (50 mL), washed with water, dried over MgSO_4_, and concentrated *in vacuo*. Then the reaction mixture was dissolved in dry THF and treated with tetrabutylammonium fluoride hydrate (TBAF, 1.80 g, 57.0 mmol) for 3 h. THF was evaporated, and the residue was extracted with EtOAc (50 mL), washed with water, dried over MgSO_4_ and concentrated *in vacuo*. The residue was purified by column chromatography (SiO_2_, EtOAc/diethyl ether 1 : 3) to yield 3b as a light-yellow oil (6.70 g, 95%). ^1^H NMR (400.13 MHz, CDCl_3_): *δ* 8.39 (d, 1H, *J* = 1.6 Hz); 7.91 (dd, 1H, *J* = 8.8, 1.6 Hz); 7.35 (d, 1H, *J* = 8.8 Hz); 7.16 (d, 1H, *J* = 3.2 Hz); 6.59 (d, 1H, *J* = 2.8 Hz); 4.20 (t, 2H, *J* = 7.2 Hz); 3.93 (s, 3H), 3.65 (t, 2H, *J* = 6.4 Hz); 1.99–1.92 (m, 2H); 1.60–1.53 (m, 2H). ^13^C NMR (100.61 MHz, CDCl_3_): *δ* 168.4, 138.6, 129.3, 128.2, 124.2, 123.0, 121.4, 110.1, 109.1, 102.9, 62.4, 52.0, 46.5, 30.0, 26.9. FT-IR (cm^−1^) *ν*_max_: 3440 (OH), 2938 (CH), 1705 (C=O). HRMS (ESI+) calcd for C_14_H_17_NO_3_, 248.1287, found 248.1286.

#### 2.2.3. Monomer 3c

To a well-stirred solution of methyl indole-5-carboxylate (1, 5.00 g, 28.5 mmol) in DMF (100 mL) was added K_2_CO_3_ (15.8 g, 4.00 eq.) and 5-bromo-1-pentanol (2c, 5.69 g, 1.20 eq.) dropwise. The reaction mixture was stirred overnight at room temperature. Afterward, the crude reaction mixture was extracted with EtOAc (50 mL), washed with water, dried over MgSO_4_, and concentrated *in vacuo*. The residue was purified by column chromatography (SiO_2_, EtOAc/diethyl ether 1 : 3) to yield 3c as a light-yellow oil (4.47 g, 60%). ^1^H NMR (400.13 MHz, CDCl_3_): *δ* 8.39 (dd, 1H, *J* = 1.6, 0.4 Hz); 7.90 (dd, 1H, *J* = 8.8, 1.6 Hz); 7.33 (d, 1H, *J* = 8.8 Hz); 7.15 (d, 1H, *J* = 3.2 Hz); 6.58 (dd, 1H, *J* = 3.2, 0.4 Hz); 4.14 (t, 2H, *J* = 7.2 Hz); 3.93 (s, 3H), 3.61 (t, 2H, *J* = 6.8 Hz); 1.92–1.85 (m, 2H); 1.63–1.55 (m, 2H); 1.44–1.36 (m, 2H). ^13^C NMR (100.61 MHz, CDCl_3_): *δ* 168.4, 138.5, 129.3, 128.2, 124.2, 122.9, 121.4, 109.1, 102.8, 62.6, 51.9, 46.6, 32.3, 30.2, 23.3. FT-IR (cm^−1^) *ν*_max_: 3461 (OH), 2945 (CH), 1705 (C=O). HRMS (ESI+) calcd for C_15_H_19_NO_3_, 262.1443, found 262.1438.

#### 2.2.4. Monomer 3d

To a well-stirred solution of methyl indole-5-carboxylate (1, 5.00 g, 28.5 mmol) in DMF (100 mL) was added K_2_CO_3_ (15.8 g, 4.00 eq.) and 6-bromo-1-hexanol (2d, 6.17 g, 1.20 eq.) dropwise. The reaction mixture was stirred overnight at room temperature. Afterward, the crude reaction mixture was extracted with EtOAc (50 mL), washed with water, dried over MgSO_4_, and concentrated *in vacuo*. The residue was purified by column chromatography (SiO_2_, EtOAc/diethyl ether 1 : 3) to yield 3d as a light-yellow oil (7.70 g, 98%). ^1^H NMR (400.13 MHz, CDCl_3_): *δ* 8.39 (d, 1H, *J* = 0.4 Hz); 7.90 (dd, 1H, *J* = 8.8, 1.2 Hz); 7.33 (d, 1H, *J* = 8.4 Hz); 7.15 (d, 1H, *J* = 3.2 Hz); 6.59 (d, 1H, *J* = 3.2 Hz); 4.14 (t, 2H, *J* = 7.2 Hz); 3.93 (s, 3H), 3.61 (m, 2H); 1.90–1.83 (m, 2H); 1.58–1.51 (m, 2H); 1.43–1.34 (m, 4H). ^13^C NMR (100.61 MHz, CDCl_3_): *δ* 168.4, 138.6, 129.3, 128.2, 124.2, 122.9, 121.4, 109.1, 102.8, 62.8, 52.0, 46.6, 32.6, 30.4, 26.9, 25.5. FT-IR (cm^−1^) *ν*_max_: 3454 (OH), 2945 (CH), 1705 (C=O). HRMS (ESI+) calcd for C_16_H_21_NO_3_, 276.1600, found 276.1598.

### 2.3. Polymer synthesis

To a 25 mL two-neck round-bottom flask equipped with mechanical stirrer and a gas inlet (connected to N_2_ or vacuum) were added monomer 3a–d (0.50 g), DBTO (5 mg) and mesitylene (5 mL) at room temperature. The reaction mixture was stirred at 180 °C for 30 minutes under N_2_. Afterward, the N_2_ flow was switched to vacuum, and the reaction was stirred at 180 °C for 6 h under vacuum. Then the reaction mixture was cooled to room temperature, dissolved in chloroform (5 mL), precipitated into diethyl ether (200 mL), and dried under vacuum to yield a off-white powder (P3a–d).

P3a (yield: 0.31 g, 71%). ^1^H NMR (400.13 MHz, CDCl_3_). *δ*, ppm: 8.36 (br. 1H), 7.89 (br. 1H), 7.39 (br. 1H), 7.20 (br. 1H), 6.61 (br. 1H), 4.31 (br. 4H), 2.30 (br. 2H). ^13^C-NMR (100.61 MHz, CDCl_3_). *δ*, ppm: 167.6, 138.8, 129.7, 128.4, 124.2, 123.1, 121.3, 109.2, 103.3, 61.4, 43.5, 29.7. FT-IR (cm^−1^) *ν*_max_: 2934 (CH), 1710 (C=O).

P3b (yield: 0.29 g, 66%). ^1^H NMR (400.13 MHz, CDCl_3_). *δ*, ppm: 8.35 (br. 1H), 7.88–7.85 (br. 1H), 7.35–7.33 (br. 1H), 7.16 (br. 1H), 6.58 (br. 1H), 4.33 (br. 2H), 4.21 (br. 2H), 2.02 (br. 2H). 1.77 (br. 2H). ^13^C-NMR (100.61 MHz, CDCl_3_). *δ*, ppm: 167.8, 138.6, 129.4, 128.2, 124.1, 123.0, 121.4, 109.2, 103.1, 63.8, 46.3, 27.1, 26.5. FT-IR (cm^−1^) *ν*_max_: 2946 (CH), 1710 (C=O).

P3c (yield: 0.34 g, 77%). ^1^H NMR (400.13 MHz, CDCl_3_). *δ*, ppm: 8.35 (br. 1H), 7.87–7.85 (br. 1H), 7.33–7.28 (br. 1H), 7.15–7.14 (br. 1H), 6.57–6.56 (br. 1H), 4.31–4.29 (br. 2H), 4.14 (br. 2H), 1.92 (br. 2H),1.80 (br. 2H), 1.48 (br. 2H). ^13^C NMR (100.61 MHz, CDCl_3_): *δ* 167.9, 138.6, 129.4, 128.2, 124.1, 122.9, 121.5, 109.1, 102.9, 64.2, 46.6, 30.0, 28.6, 23.7. FT-IR (cm^−1^) *ν*_max_: 2946 (CH), 1710 (C=O).

P3d (yield: 0.35 g, 78%). ^1^H NMR (400.13 MHz, CDCl_3_). *δ*, ppm: 8.34 (br. 1H), 7.87–7.85 (br. 1H), 7.30–7.28 (br. 1H), 7.10 (br. 1H), 6.54 (br. 1H), 4.27 (br. 2H), 4.10 (br. 2H), 1.83 (br. 2H), 1.72 (br. 2H),1.46 (br. 2H), 1.35 (br. 2H). ^13^C NMR (100.61 MHz, CDCl_3_): *δ* 168.8, 138.7, 129.5, 128.2, 124.2, 122.9, 121.1, 109.2, 102.9, 64.8, 46.6, 30.3, 28.8, 26.8, 25.9. FTIR (cm^−1^) *ν*_max_: 2957 (CH), 1710 (C=O).

### 2.4. Polymer film casting

A polymer powder (150 mg) was dissolved in 1 mL solvent at room temperature and kept stirring for 1 h to yield a clear, viscous solution. Chloroform was used as the solvent for P3a, P3b and P3d, and a 2 : 1 mixture of chloroform/hexafluoroisopropanol was used as the solvent for P3c. The resulting polymer solution was cast onto a glass Petri dish (diameter of 35 mm) and dried at room temperature for 3 days to yield a polymer film.

### 2.5. Crosslinking of polymer P3d

To a well-stirred solution of P3d (5.0 mg) in acetonitrile (2 mL) was added 3,4-dimethoxybenzaldehyde (veratraldehyde, 3 mg) and iodine (1 mg) at room temperature. The reaction was stirred at room temperature overnight under N_2_. Afterward, the solvent was evaporated, and the residue was washed with chloroform and dried over vacuum to yield the crosslinked solid P3d (5.8 mg). The swelling ratio (*Q*) of the crosslinked P3d was evaluated by measuring the mass of the material before and after being immersed in chloroform for 24 h. The *Q* value was calculated according to the equation below.^64,65^*Q* = *W*_s_/*W*_d_*W*_s_ is the weight of the swollen polymer and *W*_d_ is the weight of the crosslinked polymer in dry state.

### 2.6. Analytical methods

Nuclear magnetic resonance (NMR) measurements were carried out on a Bruker DRX 400 spectrometer at the proton frequency of 400.13 MHz and a carbon frequency of 100.61 MHz. Fourier transform infrared (FTIR) spectra were obtained with an attenuated total reflection (ATR) setup using a Bruker Alpha FT-IR spectrometer. Twenty-four scans were co-added using a resolution of 4 cm^−1^. Gel permeation chromatography (GPC) was carried out with 2xPL-Gel Mix-B LS column and OmniSEC Triple Detectors (refractive index, viscosity, and light scattering). All measurements were carried out at 35 °C at a concentration of 3 mg mL^−1^ using chloroform as the eluent, and at an elution rate of 1 mL min^−1^. Calibration was performed with polystyrene standard sample (*M*_n_ = 96 kg mol^−1^ from Polymer Laboratories). Differential scanning calorimetry (DSC) measurements were performed using a TA Instruments DSC Q2000. The samples were studied with a heating rate of 10 °C min^−1^ under nitrogen with a purge rate of 50 mL min^−1^. The sequence consisted of a heating ramp from 25 °C to 200 °C, followed by a cooling ramp to 25 °C and finally a heating ramp to 200 °C, which was employed to determine the glass transition temperature (*T*_g_). Thermogravimetric analysis (TGA) was performed with a thermogravimetric analyser TA Instruments Q500 at a heating rate of 10 °C min^−1^ under nitrogen with a purge rate of 50 mL min^−1^. Isothermogravimetric analysis was performed with a thermogravimetric analyser TA Instruments Q500 under nitrogen with a purge rate of 50 mL min^−1^. The isothermal temperatures used were 250, 275, 300, 325, 350, 375 and 400 °C. High resolution mass spectrometry (HRMS) was performed by direct infusion on a Water Xevo-G2 QTOF mass spectrometer using electrospray ionization. Dynamic mechanical analyses (DMA) were performed from 20 °C to 100 °C using a TA instrument Q800, in the cantilever bending mode at a heating rate of 3 °C min^−1^ and a frequency of 1 Hz. Samples were molded at 150 °C into rectangular bars with dimensions 35 mm (length) × 5 mm (width) × 1 mm (thickness) and cooled at room temperature. Wide angle X-ray diffraction (WAXD) measurements were performed using a Stoe STADI MP X-ray powder diffractometer under ambient conditions. Measurements were performed over 2*θ* ranges 10–100° with copper K_α_ (0.15406 nm) radiation. Based on the integrals of sharp WAXD signals, the degrees of crystallinity were quantified according to the equation below:
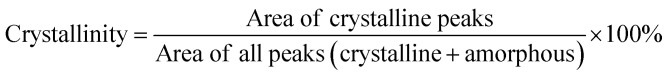


Dynamic rheology measurements were carried out using a TA Instruments Advanced Rheometer AR2000 ETC at 160 and 180 °C during 40 min. Measurements were performed using parallel plates with constant strain (2%) and oscillation (1 Hz). The specimens (15 mm diameter and 1 mm thickness) were prepared by hot-pressing the polymer powders.

## 3. Results and discussion

### 3.1. Synthesis and molecular characterization

**Scheme 1:**
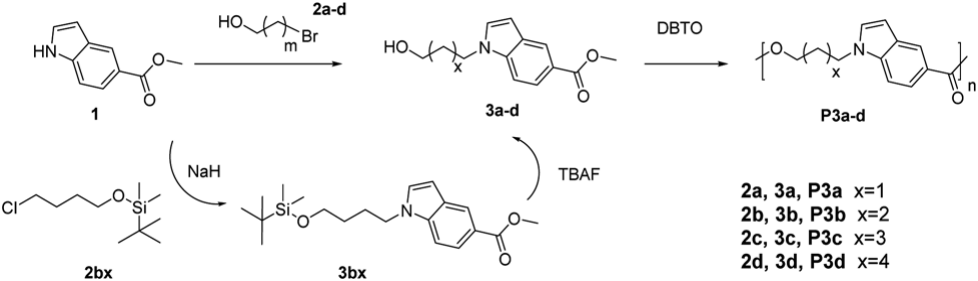
Synthesis of indole-based AB monomers (3a–d) and polyesters (P3a–d).

**Fig. 1:**
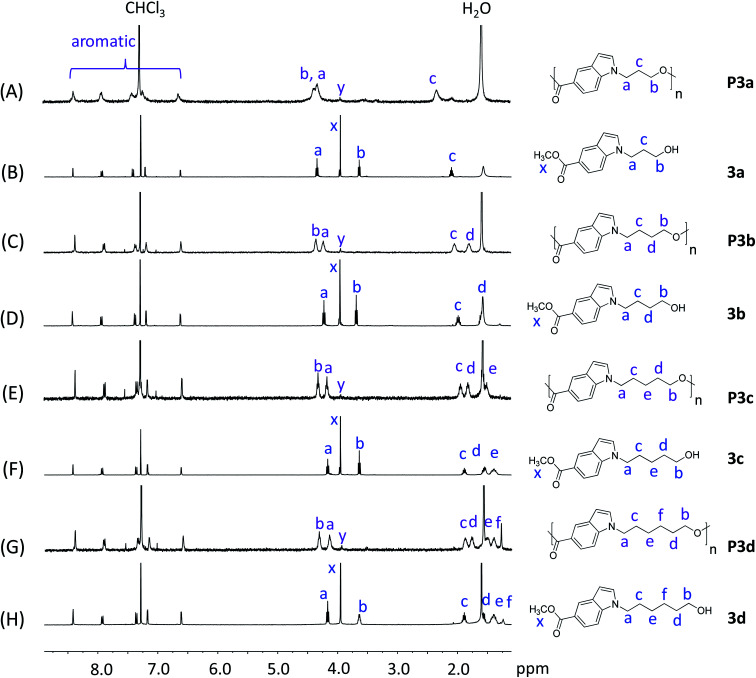
^1^H NMR spectra of monomers 3a–d (B, D, F, H) and polyesters P3a–d (A, C, E, G) in CDCl_3_. The peaks in the monomers and polymers were marked in blue and purple colors, respectively.

**Fig. 2:**
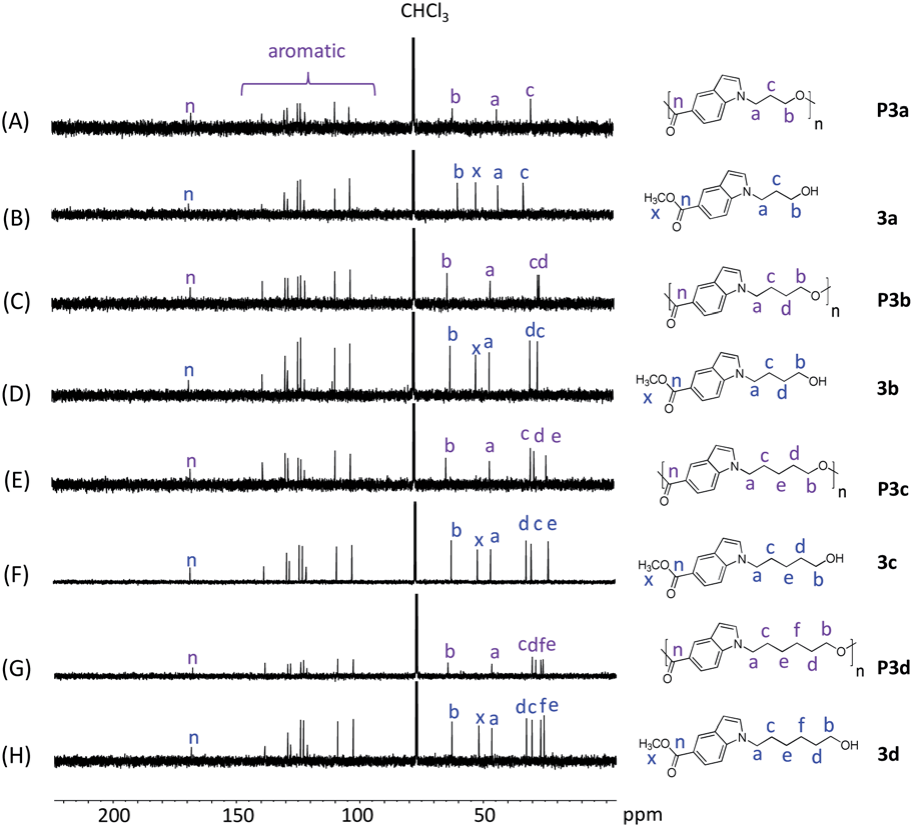
^13^C NMR spectra of monomers 3a–d (B, D, F, H) and polyesters P3a–d (A, C, E, G ) in CDCl_3_.

Monomers with both a hydroxyl and methyl carboxylate groups (AB monomers 3a–d, Scheme 1) were synthesized by the reaction of commercially available methyl indole-5-carboxylate (1) and ω-bromoalkanols (2a–d) under mild basic condition (K_2_CO_3_, room temperature). Three monomers 3a, 3c and 3d were prepared with moderate to high yields (60–98%) and high purity (according to NMR spectra, Fig. 1, 2, and S1–S24, ESI†) by this method. However, for 3b, the reaction proceeded very slowly under such conditions, and after 24 hours there was only approximately 5% of 3b in the crude reaction mixture according to the ^1^H NMR spectrum (Fig. S25, ESI†), this could be attributed to the formation of an undesired cyclic byproduct, tetrahydrofuran (THF), which was evidenced in the ^1^H NMR spectrum (Fig. S25, ESI†). The formation of THF was also reported in the literature for the synthesis of poly(butylene terephthalate) and poly(butylene succinate).^66,67^ As such, a modified synthetic protocol was used for the synthesis of 3b using *tert*-butyldimethylsilyl (TBS) protecting group. As shown in Scheme 1, a TBS-protected ω-chloroalkanol (2bx) was used to react with 1 under strong basic condition (NaH) to yield a TBS-protected monomer precursor 3bx. The crude 3bx was directly subjected to deprotection using TBAF to yield monomer 3b in 95% yield (from 1) and high purity (according to NMR analyses, Fig. 1, 2 and S7–S9, ESI†). Melt-polycondensation of monomers 3a–d was carried out according to our previously reported procedure for AB monomers with 1,3-disubstituted indole units.^58^ The polymerizations were carried out at 180 °C for 6 h under vacuum, followed by a straightforward precipitation from diethyl ether to yield off-white powders as P3a–d in decent yields (66–78%).

P3a–d and their corresponding monomers 3a–d were characterized by ^1^H NMR spectroscopy assisted with 2D-NMR spectrum (Fig. 1 and S1–S24, ESI†). First, it was evident that the ^1^H NMR spectra of P3a–d (Fig. 1A, C, E and G) showed broader peaks compared with that of the corresponding monomers 3a–d (Fig. 1B, D, F and H), which indicated the formation of polymers. Furthermore, the ^1^H NMR spectra of the monomers showed characteristic signals including CH_2_ protons close to indole (a), CH_2_ protons close to the OH group (b), protons on the aliphatic CH_2_ “bridge” (c–f), and CH_3_ protons of ester group (x). After the polymerization, the ester signal x for the monomers disappeared in the spectra of all polymers, which confirmed the monomer consumption. Note that the small signal with a slightly different chemical shift (denoted as y) was due to the methyl ester group at the chain end. All the other characteristic signals of the monomers remained. Compared to the corresponding monomer signals, signals b of the polymers showed the most significant down-field shifts (by 0.68–0.98 ppm) due to the formation of electron-withdrawing ester bonds. The second significant downfield shifts were observed for signal d in the spectra of polymers compared to that of the monomers (by 0.21–0.28 ppm), because this proton is the second closest to the ester bond (next to proton b). The chemical shifts of the other signals (a, c, e, f, and aromatic signals) did not change significantly after the polymerizations, because they are relatively far away from the reaction site (ester bonds).

The chemical structures of P3a–d were further characterized by ^13^C-NMR spectroscopy (Fig. 2). The signals for all the monomers were unambiguously assigned (Fig. 2B, D, F and H). After the polymerization, the signal x (ester CH_3_ carbon of monomers) disappeared in the ^13^C NMR spectra of all polyesters (Fig. 2A, C, E and G), confirming the success of transesterification. All the other characteristic signals remained in the spectra of polyesters, including the signals for carbonyl carbons (n), indole aromatic carbons, and the aliphatic alkylene carbons (a–f).

**Fig. 3:**
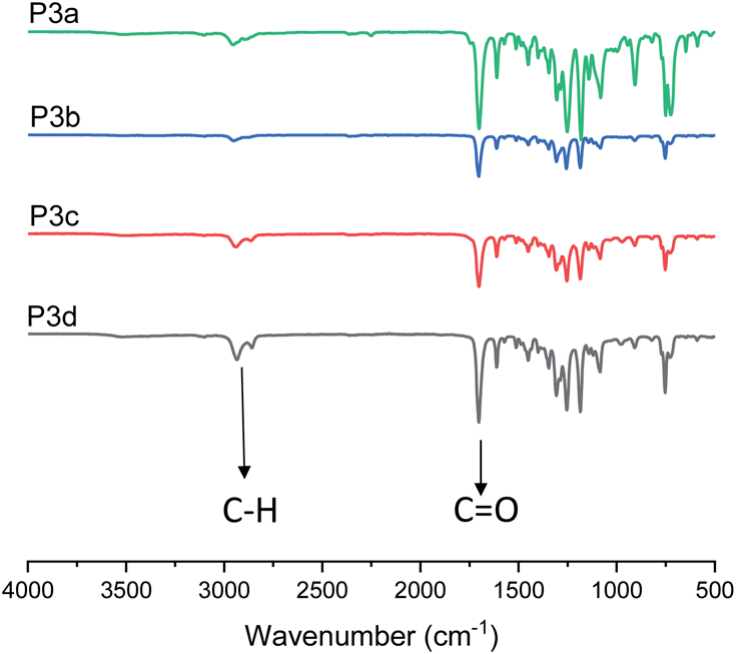
FT-IR spectra of polyesters P3a–d.

In addition, P3a–d were characterized by FT-IR spectroscopy (Fig. 3). The characteristic C–H and C=O stretching bands for the polyesters were clearly observed at 2940 and 1710 cm^−1^, respectively. In the FT-IR spectra of the corresponding monomers (Fig. S26A, ESI†), an O–H stretching (3150–3650 cm^−1^) band was observed, which completely disappeared in the spectra of all polymers. This further confirmed the consumption of monomers. A close comparison between the FT-IR spectra of monomer 3c and polymer P3c was shown as an example in Fig. S26B.†

### 3.2. Thermal and dynamic mechanical properties

**Fig. 4:**
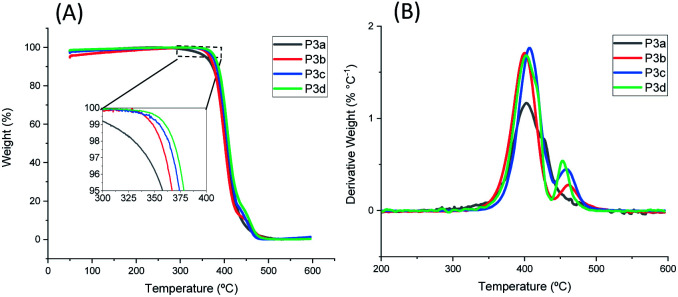
TGA (A) weight loss curves and (B) derivative curves of polyesters P3a–d.

The thermal stability of P3a–d was investigated by TGA measurements in nitrogen. As shown in Fig. 4 and Table 1, all the polyesters were thermally stable with *T*^95^_d_ > 350 °C. The *T*^95^_d_ values increased as the increased length of alkylene units (*T*^95^_d_ = 358, 367, 374, 378 °C for P3a–d), which could be related to the increased molecular weight of the repeating unit and thus decreased relative content of labile ester bonds. This observed trend is consistent with that for the previously reported series of polyesters with 1,3-disubstituted indoles (chemical structures of P4a–d see Fig. S27,†*T*^95^_d_ values: 335, 337, 343, 349 °C, respectively).^58^ Furthermore, the polyesters with 1,5-disubstituted indole units (P3a–d) showed considerably higher *T*^95^_d_ values (∼29–31 °C higher) compared to the corresponding polyesters with 1,3-disubstituted indoles (P4a–d). Since the corresponding members of the two series of polyesters (P3a–d and P4a–d) showed comparable molecular weights (P3a, P3c showed slightly higher, while P3b and P3d showed slightly lower molecular weights compared to their counterparts in P4a–d series), the observed enhanced thermal stability of polyesters P3a–d series was reasonably attributed to the molecular structure of 1,5-disubstituted indole units in the backbones. To further verify this, TGA measurements of 1,5-disubstituted monomers 3a–d and the corresponding 1,3-disubstituted monomers 4a–d were conducted and compared (Fig. S28, ESI†). As a result (Table S1, ESI†), *T*^95^_d_ and *T*_d_ values of 3a–d were higher than that of corresponding monomers 4a–d with the same number of CH_2_ units. This confirmed that the enhanced thermal stability of P3a–d (compared to the corresponding P4a–d with the same number of CH_2_ units) could be attributed to the higher thermal stability of the 1,5-disubstituted monomeric units under the TGA conditions.

**Table 1:** Molecular characterization and thermal properties of P3a–d. *M*_n_, *M*_w_, and PDI were determined by GPC. *T*^95^_d_ (temperature for 5% weight loss) was measured by TGA. The yield is the isolated polymer yield (in %)

Polymers	*M*_n_ (g mol^−1^)	*M*_w_ (g mol^−1^)	PDI	*T*_g_ a (°C)	*T*_g_ b (°C)	*T* ^95^_d_ (°C)	Yield (%)
P3a	12 800	43 300	3.4	80	77	349	71
P3b	9900	16 500	1.7	75	69	343	66
P3c	14 700	39 800	2.7	64	61	337	77
P3d	13 700	30 000	2.2	57	51	335	78

a *T*_g_ was measured by the DSC second heating curves.

b *T*_g_ was taken as the peak values of the loss modulus curves measured by DMA.

To gain further insight into the thermal stability of the indole-based polyesters, we performed isothermal TGA measurements on P3d and other two previously synthesized polyesters with 1,3-disubstituted indole units (P4d and P5d, chemical structures shown in Fig. S27, ESI†),^50,58^ both all of which are the most thermally stable members of their corresponding series according to their *T*^95^_d_ values (TGA). The isothermal TGA measurements were performed at seven elevated temperatures (*i.e.*, 250, 275, 300, 325, 350, 375, and 400 °C). As shown in Fig. S29 and Table S2 (ESI†), P3d showed a lower weight loss rate compared with P4d and P5d at all the temperatures measured, which indicated its enhanced long-term thermal stability.

The thermal stability of P3d, P4d and P5d under mechanical strain was evaluated by time-sweeping rheology measurements at two mildly high temperatures (160 °C and 180 °C) at a frequency of 1 Hz using a constant strain of 2%. This ensured that the measurements were performed within the linear viscoelastic region. As shown in Fig. S30 (ESI†), the melt shear storage modulus of P5d at both temperatures showed an increasing trend over time, which indicated crosslinking and gel formation.^68^ Such phenomenon was not observed for P3d and P4d at 160 °C, indicating the stability of both polymers under such processing conditions. At higher temperature (180 °C), both P3d and P4d showed a slight increase of the shear storage moduli, indicating some extent of crosslinking and gelation.

**Fig. 5:**
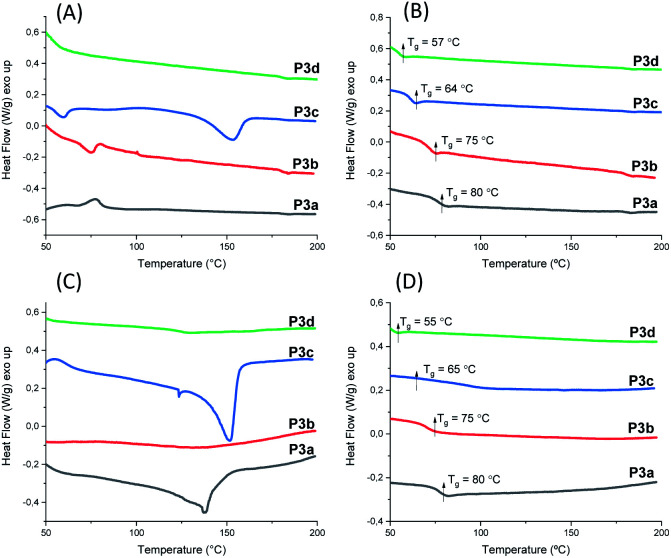
DSC (A) first (A) and second (B) heating curves of P3a–d powders, and first (C) and second (D) heating curves of P3a–d solution-cast films.

The thermal behavior of polyesters P3a–d was investigated by DSC measurements. Both powders (precipitated from chloroform/ether solution) and films (cast from chloroform solution, see later section) of P3a–d were measured. The glass transition was clearly observed in the second heating curves of both the films (Fig. 5B) and the powders (Fig. 5D) and the *T*_g_ values were consistent (Table 1). The *T*_g_ values for P3a–d decreased as the increased length of aliphatic alkylene units in the backbone (*T*_g_ ≈ 80, 75, 64 and 57 °C for P3a–d, respectively), which was consistent with the increased backbone flexibility. This trend was also observed for the previously reported indole-based polyesters with 1,3-disubstitution pattern (P4a–d, P5a–d),^58^ as well as polyesters with other bio-sourced aromatic units (*e.g.* furan, vanillic acid).^69–72^

DSC results also provided valuable information regarding the crystallinity of P3a–d as solvent-cast films and solution-precipitated powders. As shown in Fig. 5A, the powder of P3c showed a melting endotherm in its first heating curve (peak value at 153 °C). However, no melting endotherm was observed during the second heating cycle (Fig. 5B). This indicated that P3c crystallized from solution but not from melt during the DSC measurements. All the other polymer powders did not crystalize from solution or melt. For solution-cast films, melting endotherms were observed during the first heating cycle of P3a and P3c (*T*_m_ = 138 and 151 °C for P3a and P3c respectively, Fig. 5C), which indicated that they crystalized from solution. Like powders, no melting endotherms were observed during the second heating cycle (Fig. 5D), which indicated that P3a and P3c did not crystalize from melts under the DSC conditions. This behavior could be attributed to the higher viscosity of polymer melts compared to their solutions, which caused slower crystallization rate of the polymers in molten state. Similar observation was also reported for other polyesters with rigid cyclic sugar-based units.^73,74^

**Fig. 6:**
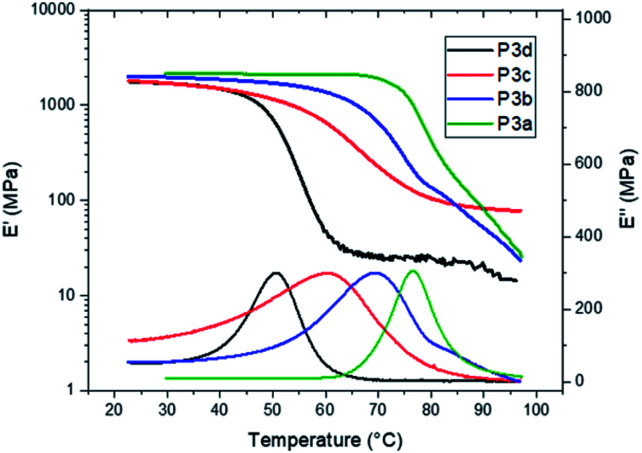
DMA storage (*E*′, upper) and loss (*E*′′, lower) modulus curves of polyesters P3a–d.

The *T*_g_ values of P3a–d were independently confirmed by DMA measurements (Fig. 6). The peak values on the loss modulus (*E*′′) curves were taken as the *T*_g_ values (77, 69, 61 and 51 °C for P3a–d, respectively, Fig. 6 and Table 1), which were consistent with the corresponding values measured by DSC (Δ*T*_g_ = 3–6 °C). The storage moduli (*E*′) at glassy plateau (25 °C) of P3a–d showed a decreasing trend as the increased length of alkylene unit (2009, 1969, 1777 and 1752 MPa, respectively), which was consistent with the trend reported for other indole-based polyesters with 1,3-disubstitution pattern.^50,58^ The storage moduli (*E*′) at glassy plateau (25 °C) of P3a–d were higher than the corresponding values for the polyesters with 1,3-disubstitution pattern (P4a–d and P5a–d) under identical measurement conditions, but lower than the corresponding values of bottle-grade PET and PET-like copolyester Akestra™ (from Perstorp AB) under the same measurement conditions (∼3000 MPa).^58^

### 3.3. Solution-casting films and WAXD

**Fig. 7:**
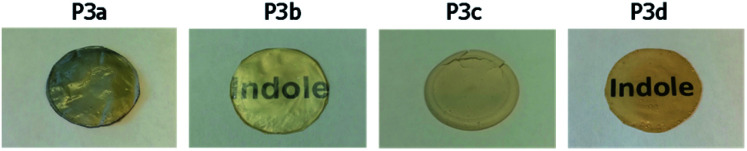
Solution-casting films of polyesters P3a–d.

P3a–d were successfully cast into thin films according to a solution casting protocol using chloroform (for P3a, P3b and P3d) or chloroform/HFIP mixture (for P3c) as the solvent.^75^ UV-vis spectra of P3a–d solutions (Fig. S31, ESI†) showed typical indole absorbance band at ∼280 nm. As shown in Fig. 7, the films of P3a and P3c were opaque, while the other two films for P3b and P3d were translucent. This was consistent with the observed crystallinity of P3a and P3c and amorphous nature of P3b and P3d in DSC first heating cycle discussed earlier.

**Fig. 8:**
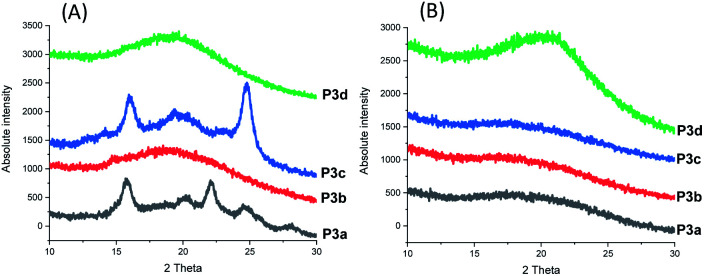
WAXD patterns of the solution-casting films (A) and powders (B) of P3a–d.

The crystallinity of P3a–d solution-cast films and powders were confirmed by WAXD (wide angle X-ray diffraction) analysis (Fig. 8). For P3b and P3d films, no sharp signal was observed in their WAXD curves (only a broad signal centered at 2*θ* = 19.2°), which confirmed their amorphous state. In the WAXD pattern of P3a film (Fig. 8A), 5 sharp signals (2*θ* = 15.7, 20.1, 22.1, 24.5 and 28.0°) were observed, which corresponded to the different planes in its crystalline structures. For P3c, four sharp signals (2*θ* = 14.2, 15.9, 19.3 and 24.8°) were observed. Based on the integrals of their sharp WAXD signals, the degrees of crystallinity were quantified as 71% and 69% for P3a and P3c, respectively (Fig. S32, ESI†).

Interestingly, it was the polyesters with odd numbers of methylene units per repeating unit that showed higher crystallinity (*i.e.*P3a, P3c with 3 or 5 CH_2_ units per repeating unit, respectively). This was different from the observation for the previously reported polyester series with 1,3-disubstituted indoles (P4a–d), which showed increased crystallinity upon the increased number of methylene units per repeating unit (*i.e.*P4c and P4d showed degrees of crystallinity as 58 and 77%, respectively, while P4a-b were amorphous).^58^

Finally, P3a–d in their powder form (precipitated from chloroform/ether) were also measured by WAXD for comparison. As shown in Fig. 8B, no sharp signal was observed for any of the measured polymer powders, which indicated that no crystallization occurred during the precipitation from chloroform/ether at room temperature or during storage at room temperature. This result also indicated that the small crystallinity of the P3c powder observed in DSC measurements (Fig. 5A) was insignificant.

### 3.4. Crosslinking with biobased aldehyde

**Scheme 2:**
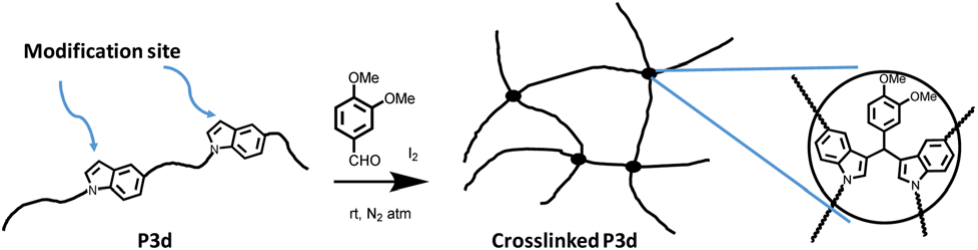
Crosslinking of P3d with 3,4-dimethoxybenzaldehyde (veratraldehyde).

**Fig. 9:**
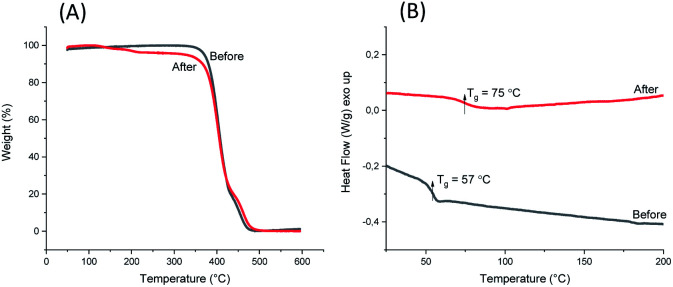
TGA weight loss curves (A) and DSC second heating curves (B) of P3d before and after aldehyde crosslinking.

1,5-Disubstituted indole unit has no substituent at the 3 position, which is the most electrophilic position of indole. This indicates that the new polyesters P3a–d may be conveniently modified on the indole rings at the 3 position, enabling quick chemical modification. In this work, we performed a crosslinking reaction of P3d (Scheme 2) with lignin-based 3,4-dimethoxybenzaldehyde^76^ by a straightforward Friedel–Crafts reaction. After the crosslinking of P3d in the solution, an insoluble gel was formed, which was dried and subjected to TGA, DSC and FTIR measurements (Fig. 9). According to TGA results, thermal stability of P3d remained almost unchanged after crosslinking (Fig. 9A). DSC results indicated that the *T*_g_ value increased from 57 to 75 °C after crosslinking (Fig. 9B), showing enhanced thermal performance. FT-IR spectra of P3d remained almost unchanged after crosslinking (Fig. S33, ESI†). The swelling ratio of the crosslinked P3d was determined as ∼6.2, after the pre-dried sample was immersed in chloroform at room temperature for 24 h. This preliminary investigation indicated the potential for enhancing the performance of polyesters with 1,5-disubstituted indole units in the future.

## 4. Conclusions

A new series of polyesters with 1,5-disubstituted indole units was synthesized and compared to our previously reported polyesters with 1,3-disubstitution pattern, aiming to provide fundamental insight into the impact of di-substitution pattern on the physical properties and processing characteristics. According to the DSC and WAXD results and visual inspection on clarity, the solution-cast films of the obtained polyesters showed crystallinity when odd numbers (3 or 5) of methylene units were present in each backbone repeating units. Those polyesters with even numbers (4 or 6) of methylene units per repeating unit were amorphous. TGA results revealed enhanced thermal stability of the new series of polyesters compared to the previously reported polyesters with 1,3-disubstituted indole units. Melt-rheology measurements confirmed the suitable processability of the new polyesters. Finally, crosslinking at the 3 position of indole units in the new polyester by bio-based aldehyde showed further enhanced thermal properties, which may have the potential to broaden the application range of these new polyesters. Future investigations will be directed toward optimization of the polymerization and processing conditions, which will facilitate investigation on other important material properties, *e.g.*, mechanical and barrier properties.

## Author contributions

PW performed the synthesis and characterization of all the polyesters. BZ conceived and advised on the project. PW and BZ wrote the paper.

## Conflicts of interest

There are no conflicts to declare.

## Supplementary Material

RA-011-D1RA02197D-s001
