# Supplementary material for: Sustainable aromatic polyesters with 1,5-disubstituted indole units
Source: RSC Adv. 2021 May 5;11(27):16480–9. doi: 10.1039/d1ra02197d (PMC9031847; doi:10.1039/d1ra02197d)
Supplement: RA-011-D1RA02197D-s001 [file RA-011-D1RA02197D-s001.pdf]

Electronic Supplementary Material (ESI) for RSC Advances.
This journal is © The Royal Society of Chemistry 2021

Supporting Information

Sustainable aromatic polyesters with 1,5-disubstitutied indole units

*Ping Wang[1], Baozhong Zhang[1]**

[1] Centre of Analysis and Synthesis, Department of Chemistry, Lund University, P.O. Box 124, 22100 Lund, Sweden

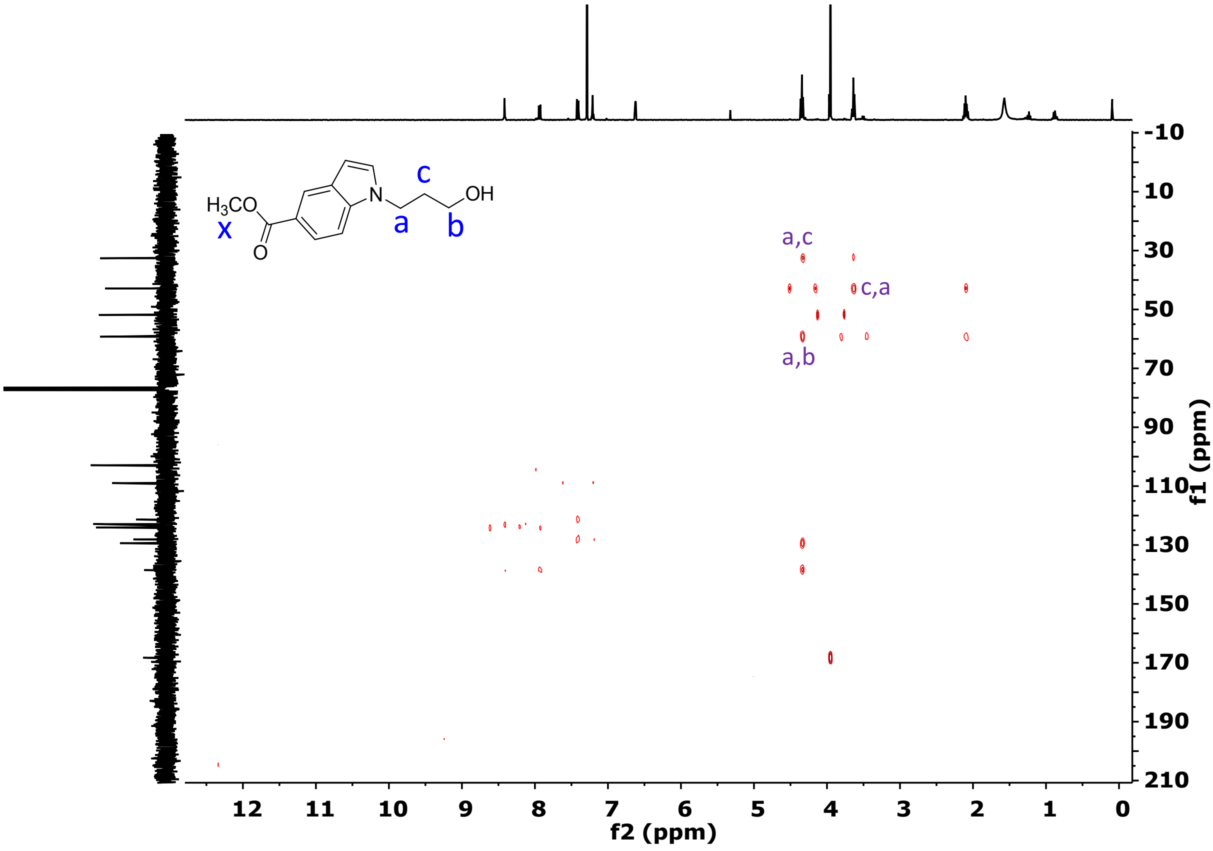

Figure S1 HMBC NMR spectrum of monomer **3a** in $CDCl_3$.

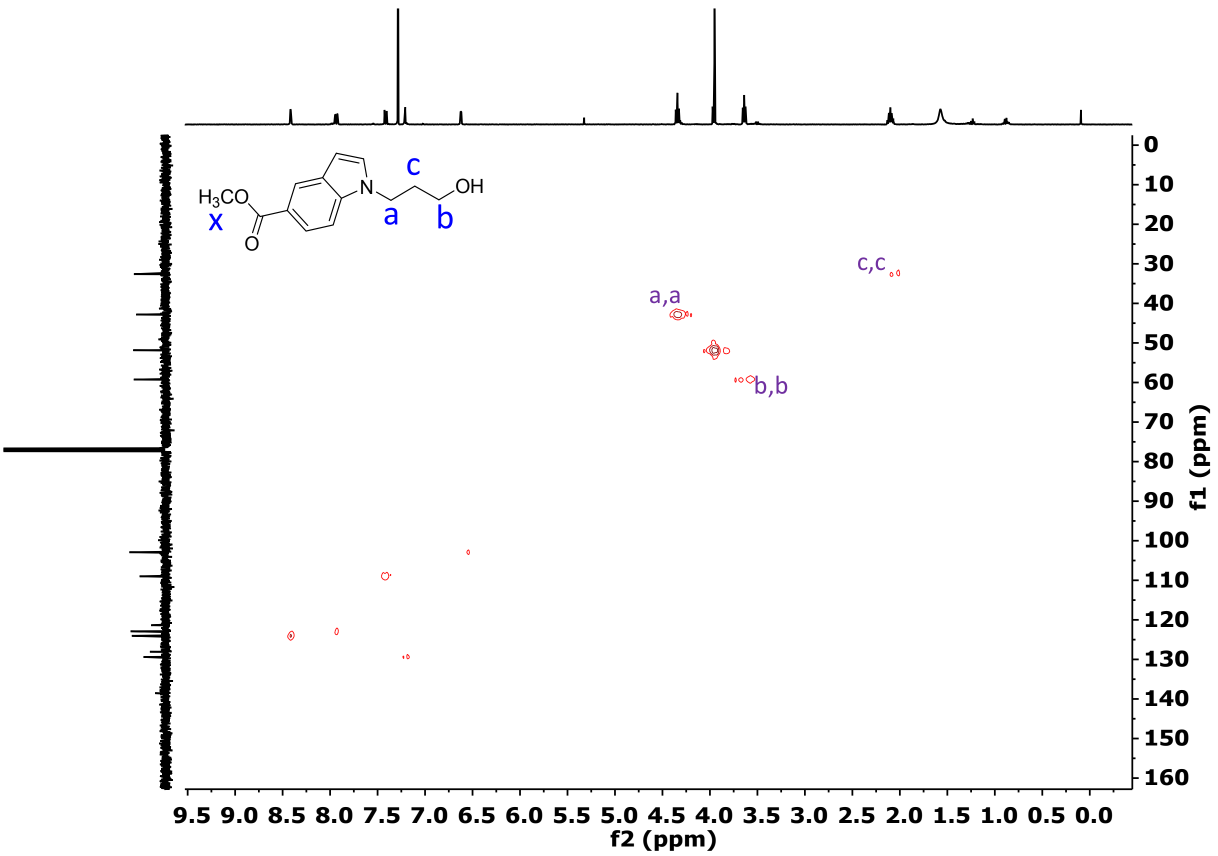

Figure S2 HMQC NMR spectrum of monomer **3a** in $CDCl_3$.

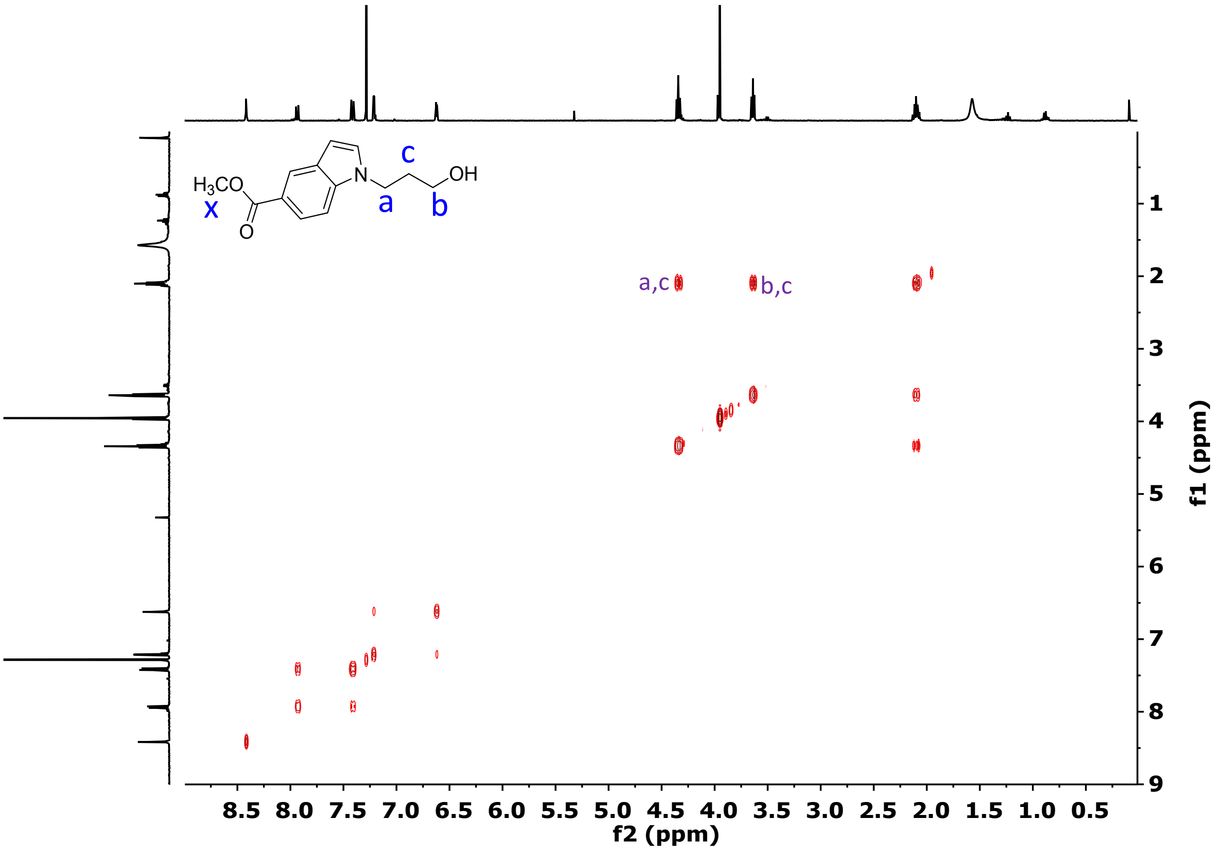

Figure S3 COSY H NMR spectrum of monomer **3a** in $CDCl_3$.

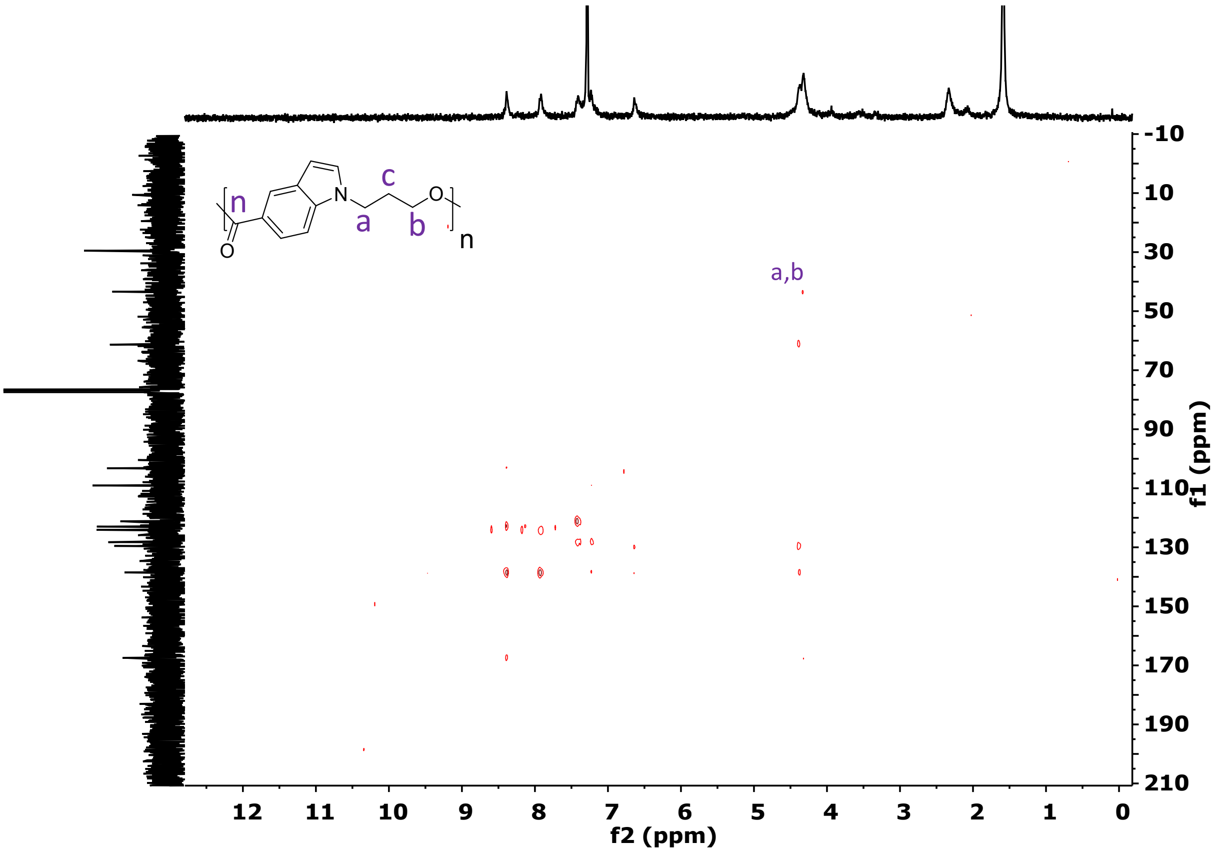

Figure S4 HMBC NMR spectrum of polymer **P3a** in $CDCl_3$.

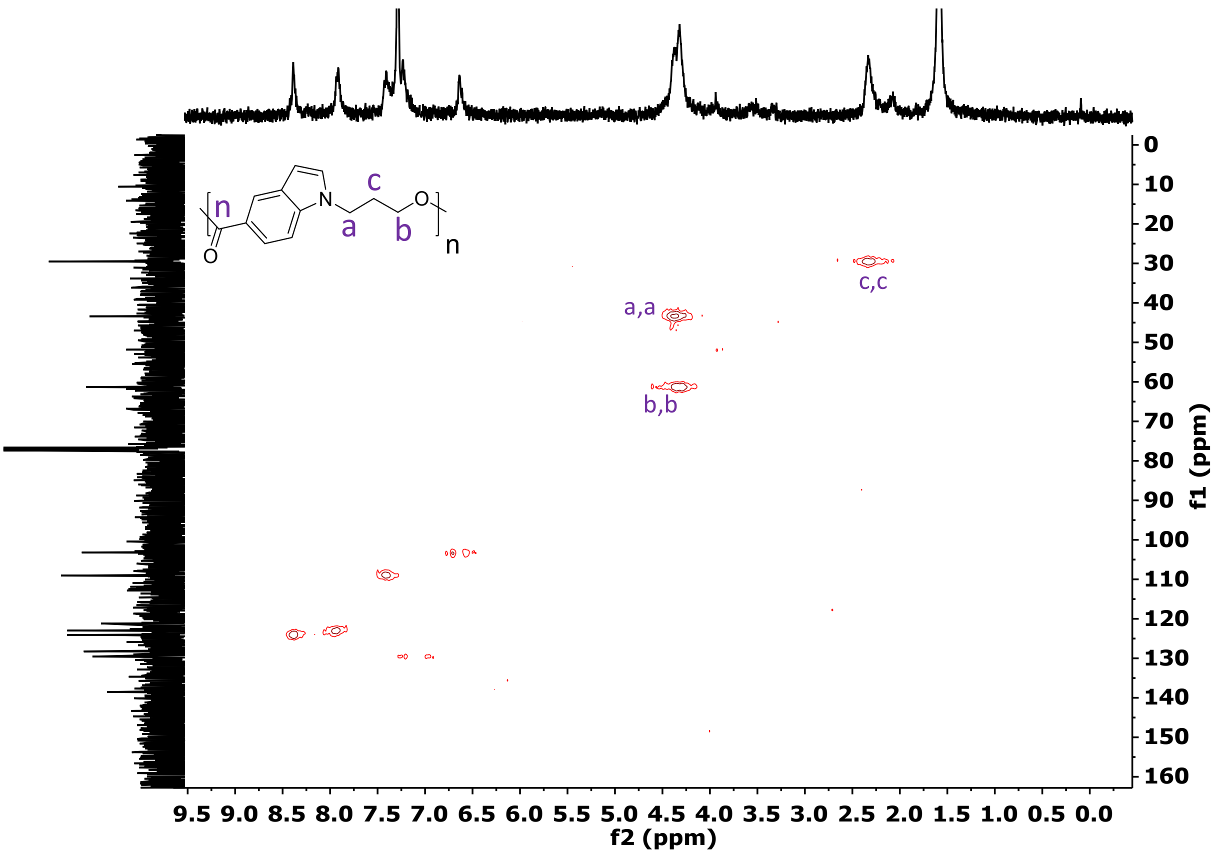

Figure S5 HMQC NMR spectrum of polymer **P3a** in $CDCl_3$.

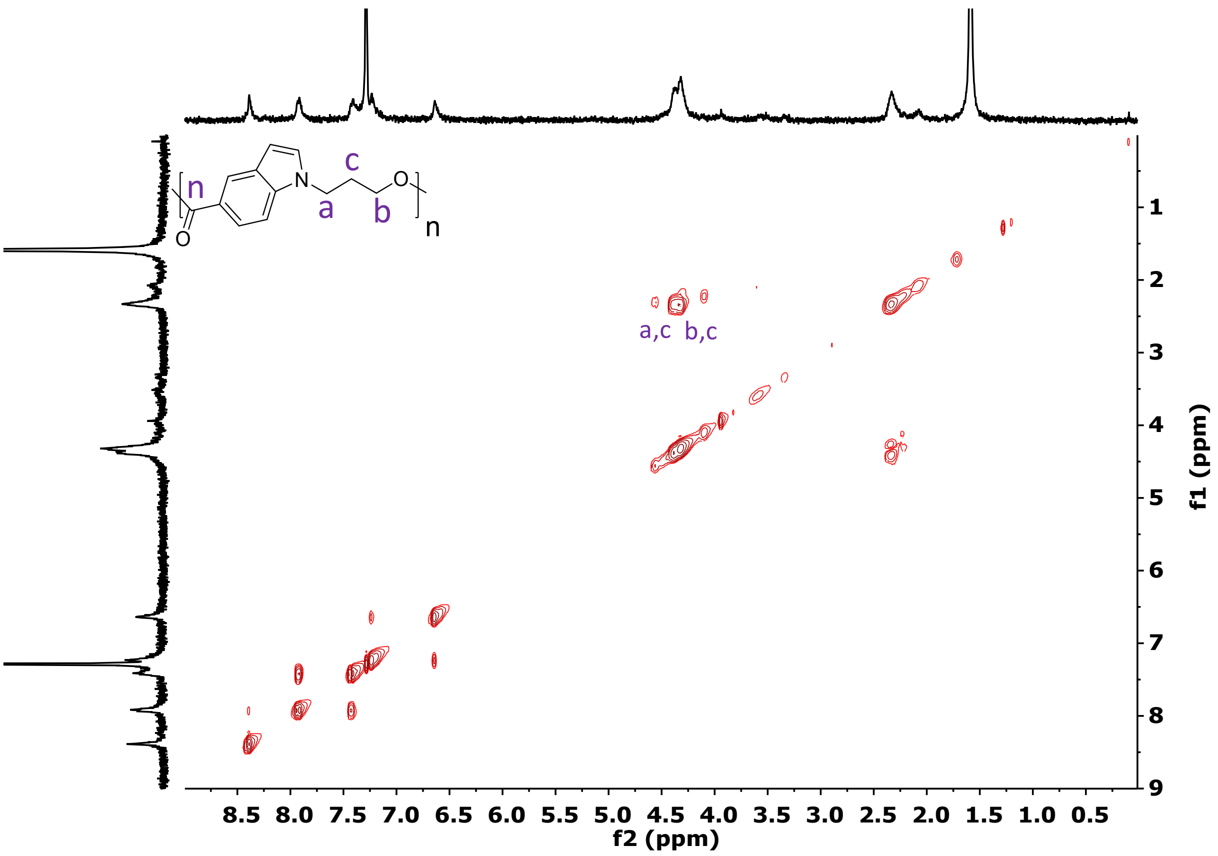

Figure S6 COSY NMR spectrum of polymer **P3a** in $CDCl_3$.

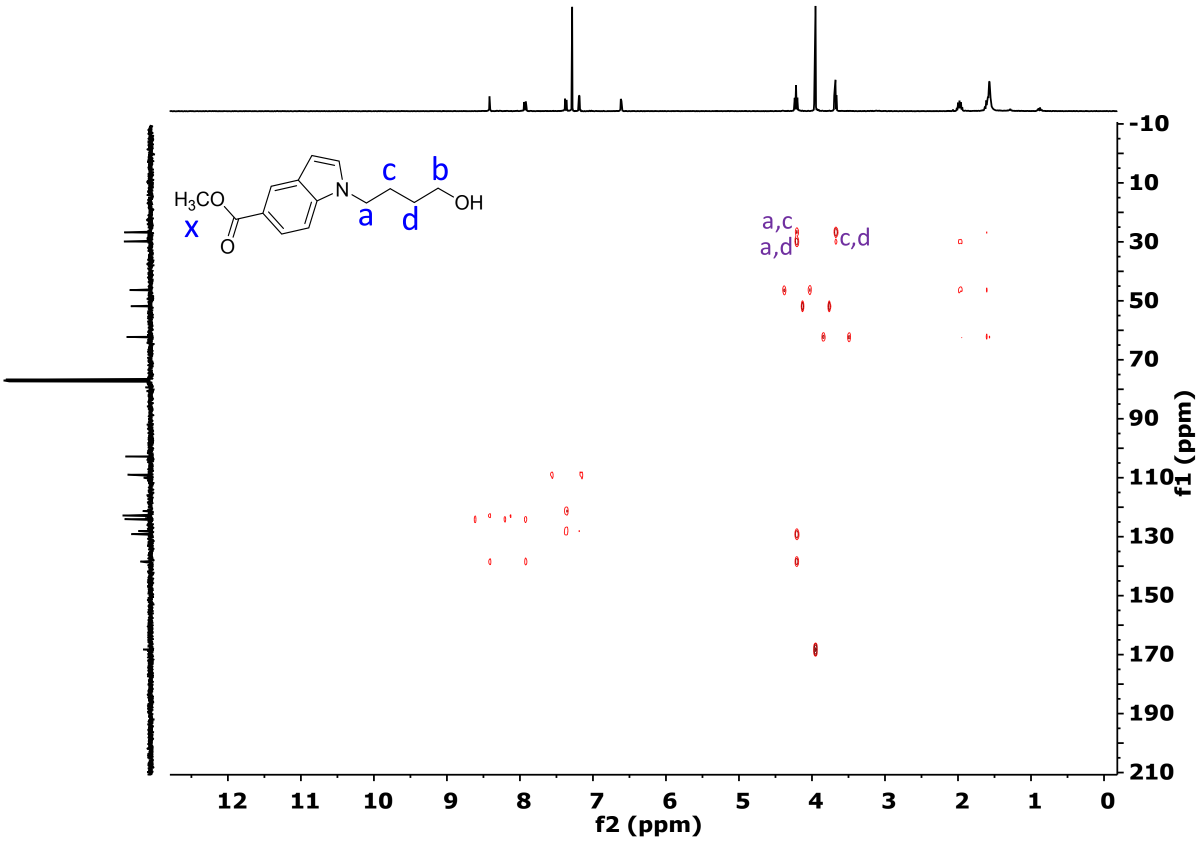

Figure S7 HMBC NMR spectrum of monomer **3b** in $CDCl_3$.

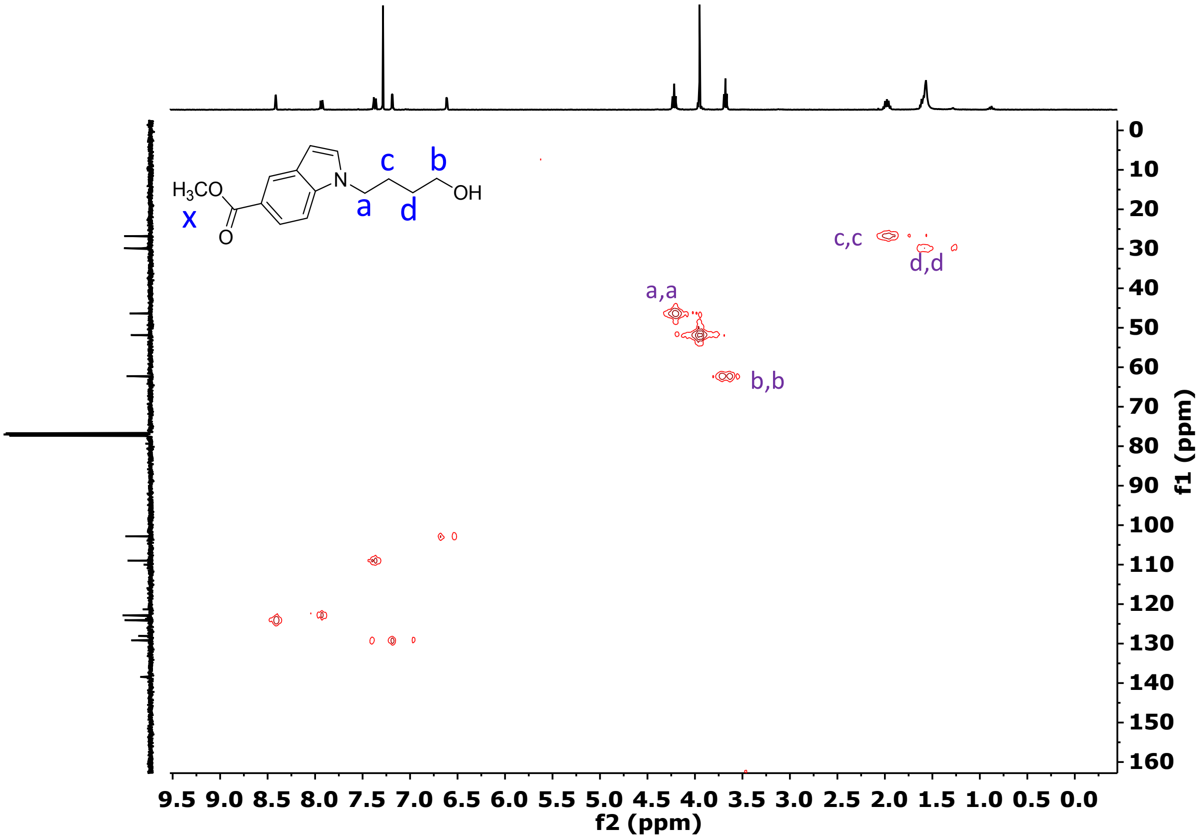

Figure S8 HMQC NMR spectrum of monomer **3b** in $CDCl_3$.

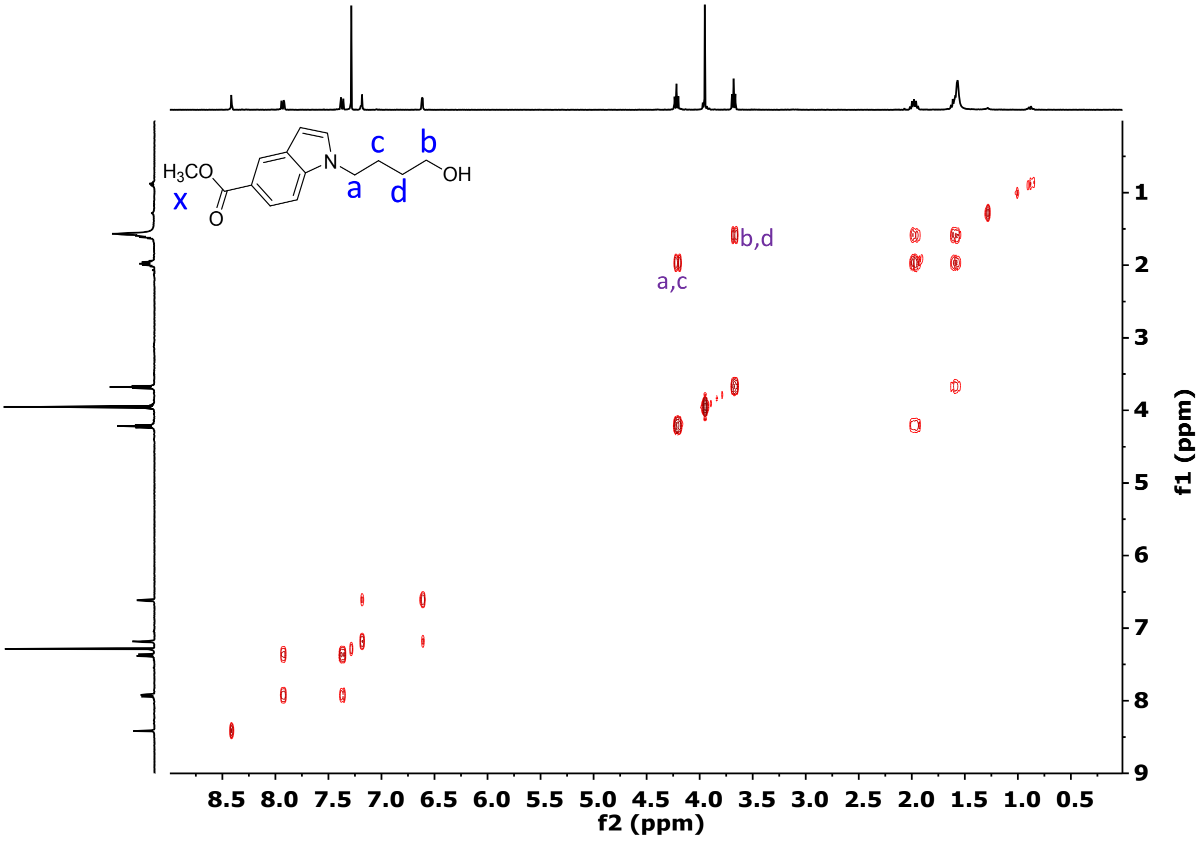

Figure S9 COSY NMR spectrum of monomer **3b** in $CDCl_3$.

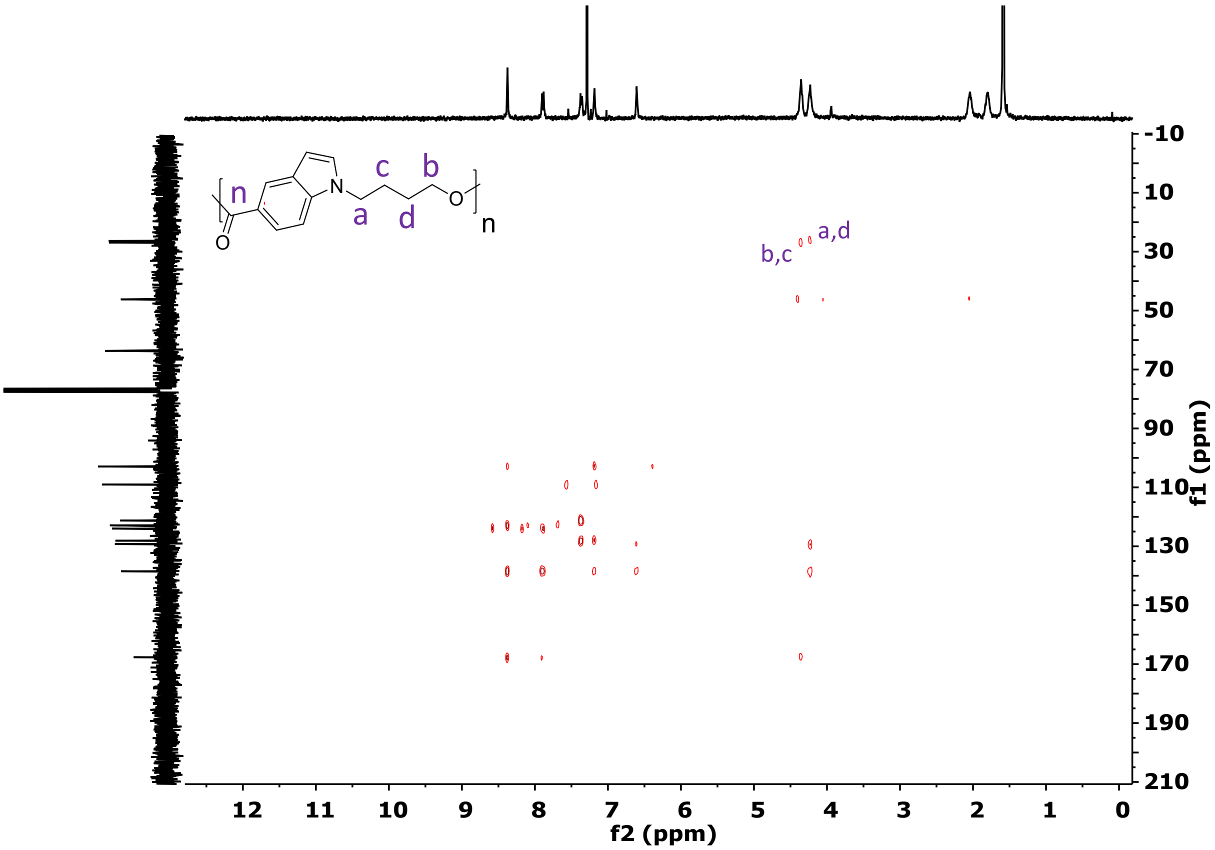

Figure S10 HMBC NMR spectrum of polymer **P3b** in $CDCl_3$.

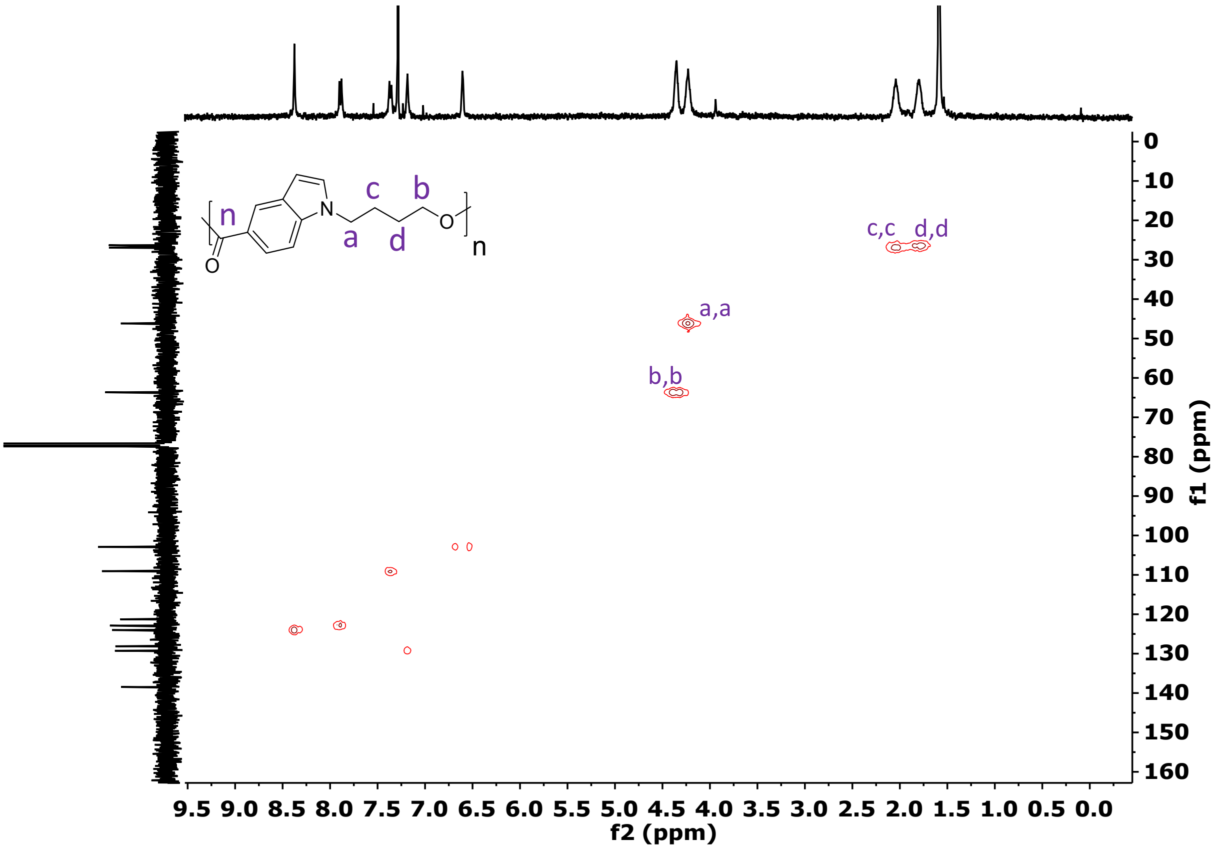

Figure S11 HMQC NMR spectrum of polymer **P3b** in $CDCl_3$.

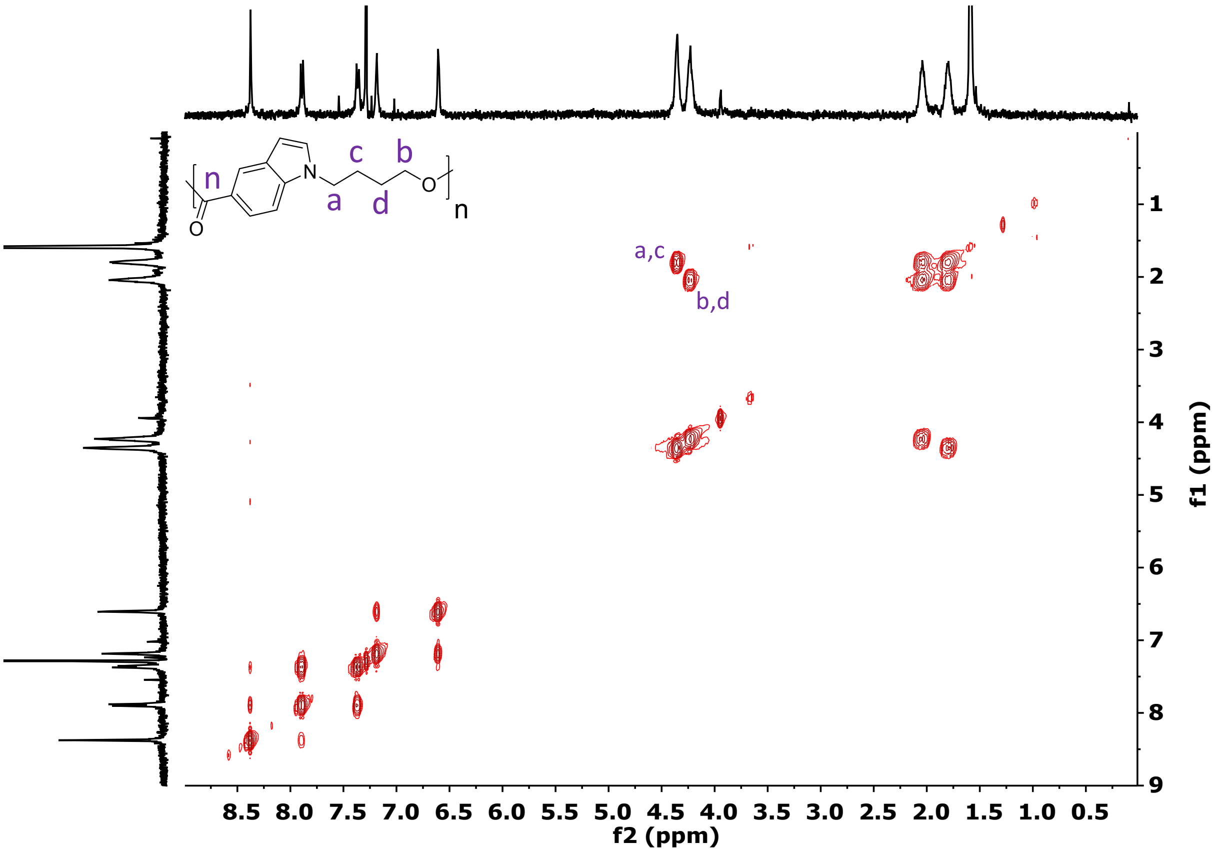

Figure S12 COSY NMR spectrum of polymer **P3b** in $CDCl_3$.

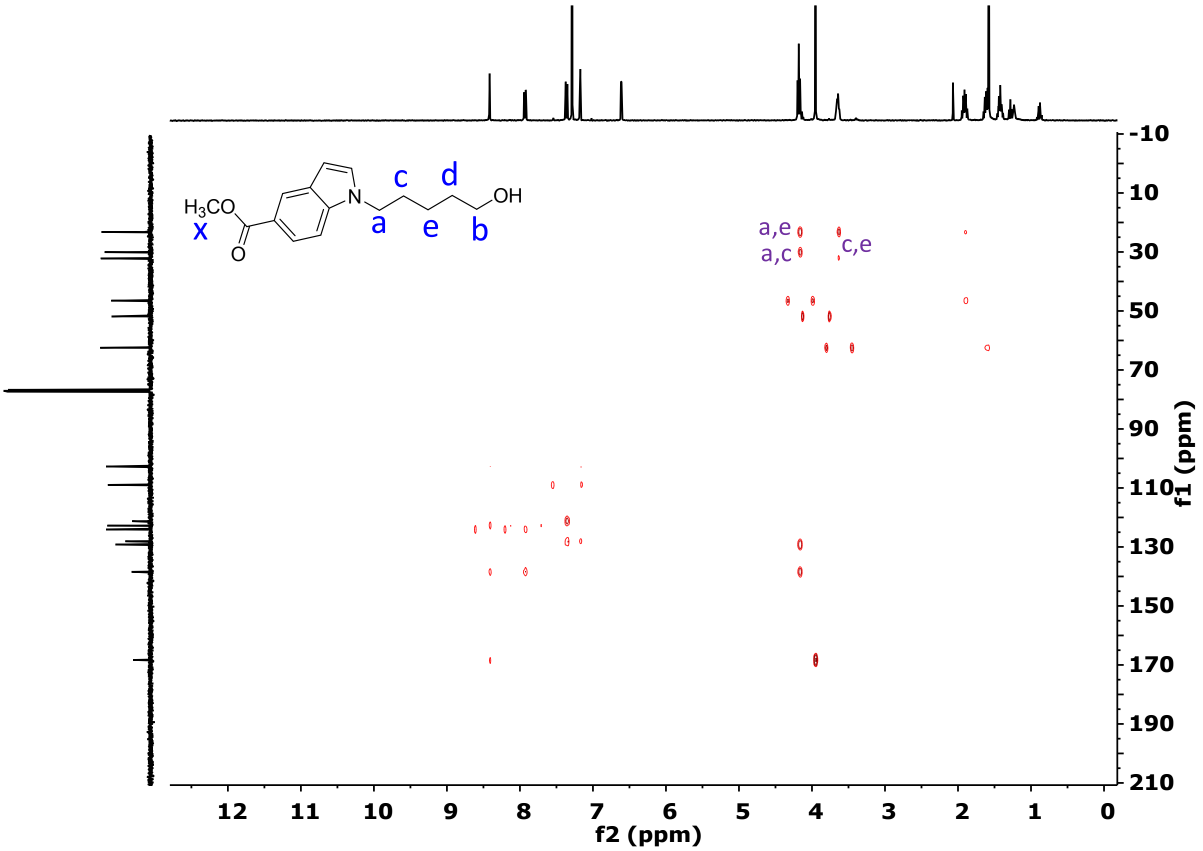

Figure S13 HMBC NMR spectrum of monomer **3c** in $CDCl_3$.

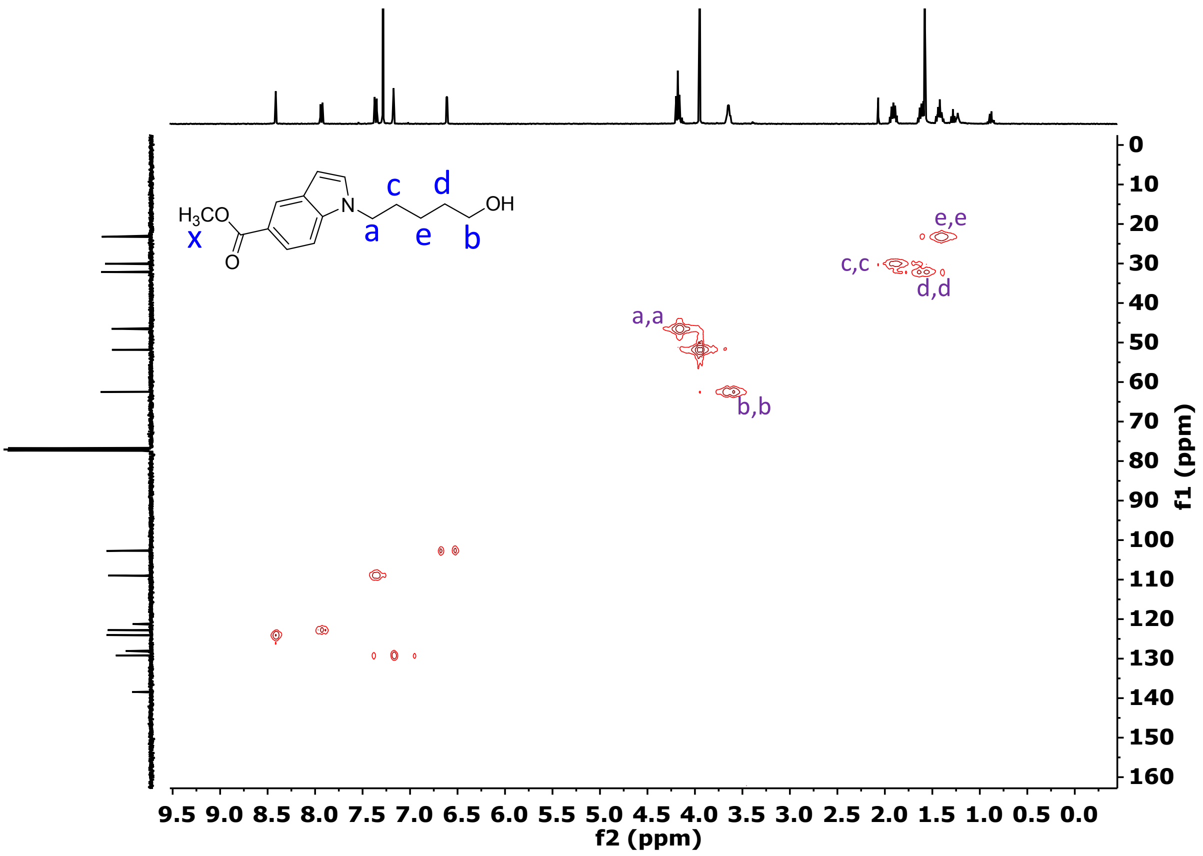

Figure S14 HMQC NMR spectrum of monomer **3c** in $CDCl_3$.

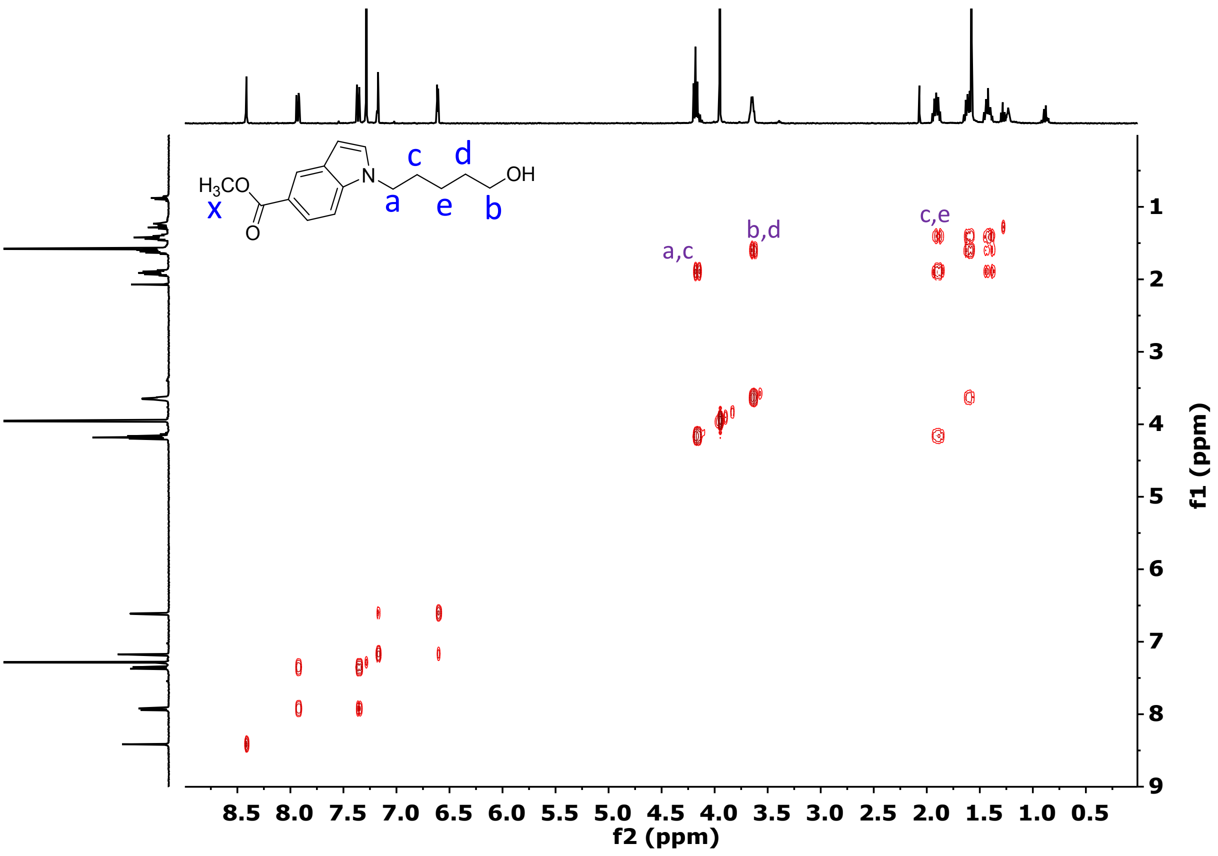

Figure S15 COSY NMR spectrum of monomer **3c** in $CDCl_3$.

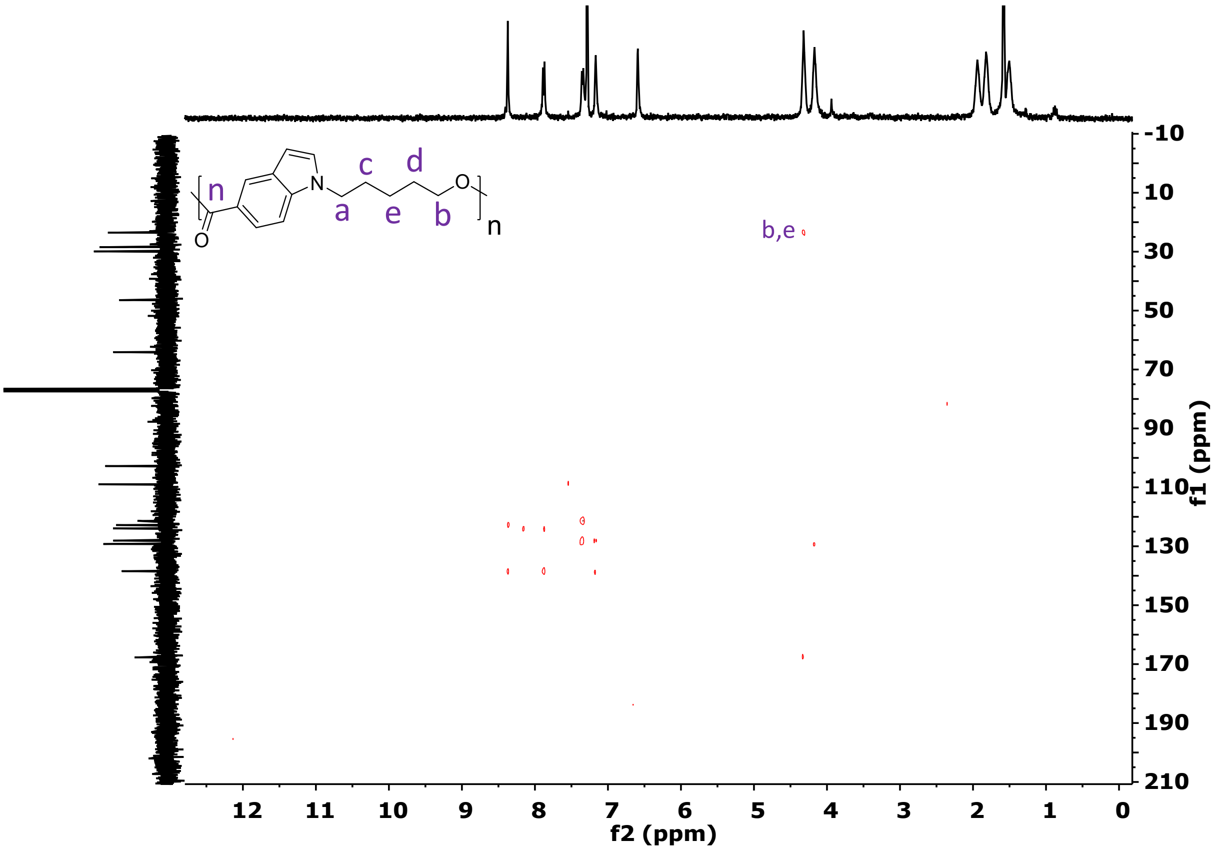

Figure S16 HMBC NMR spectrum of polymer **P3c** in $CDCl_3$.

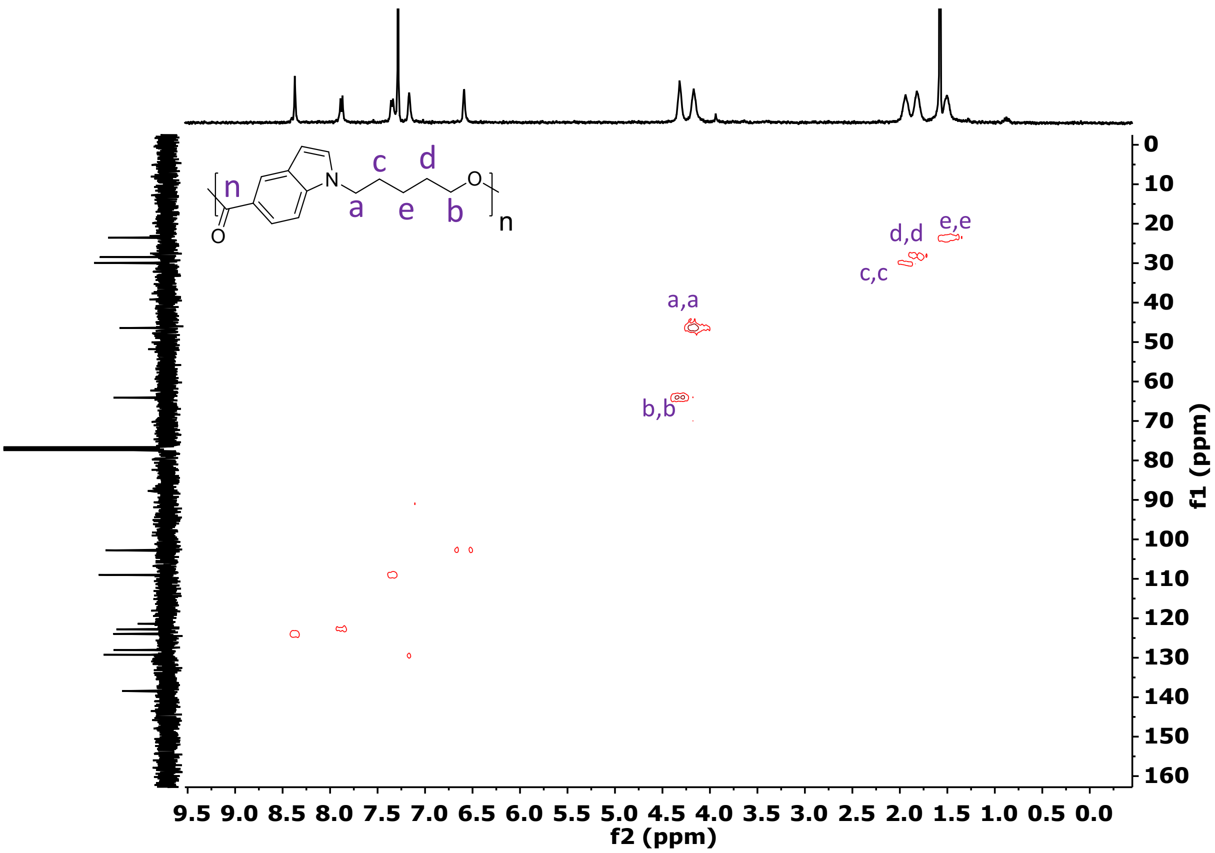

Figure S17 HMQC NMR spectrum of polymer **P3c** in $CDCl_3$.

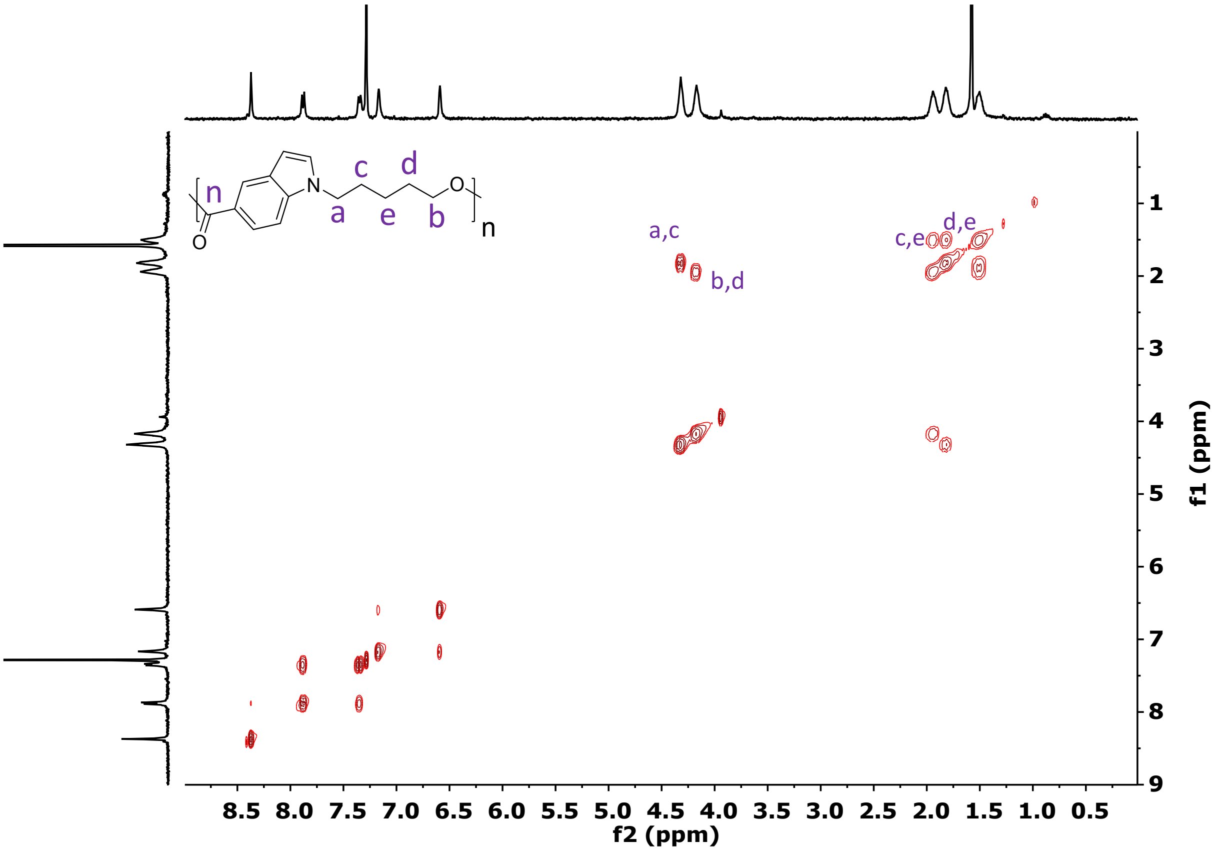

Figure S18 COSY NMR spectrum of polymer **P3c** in $CDCl_3$.

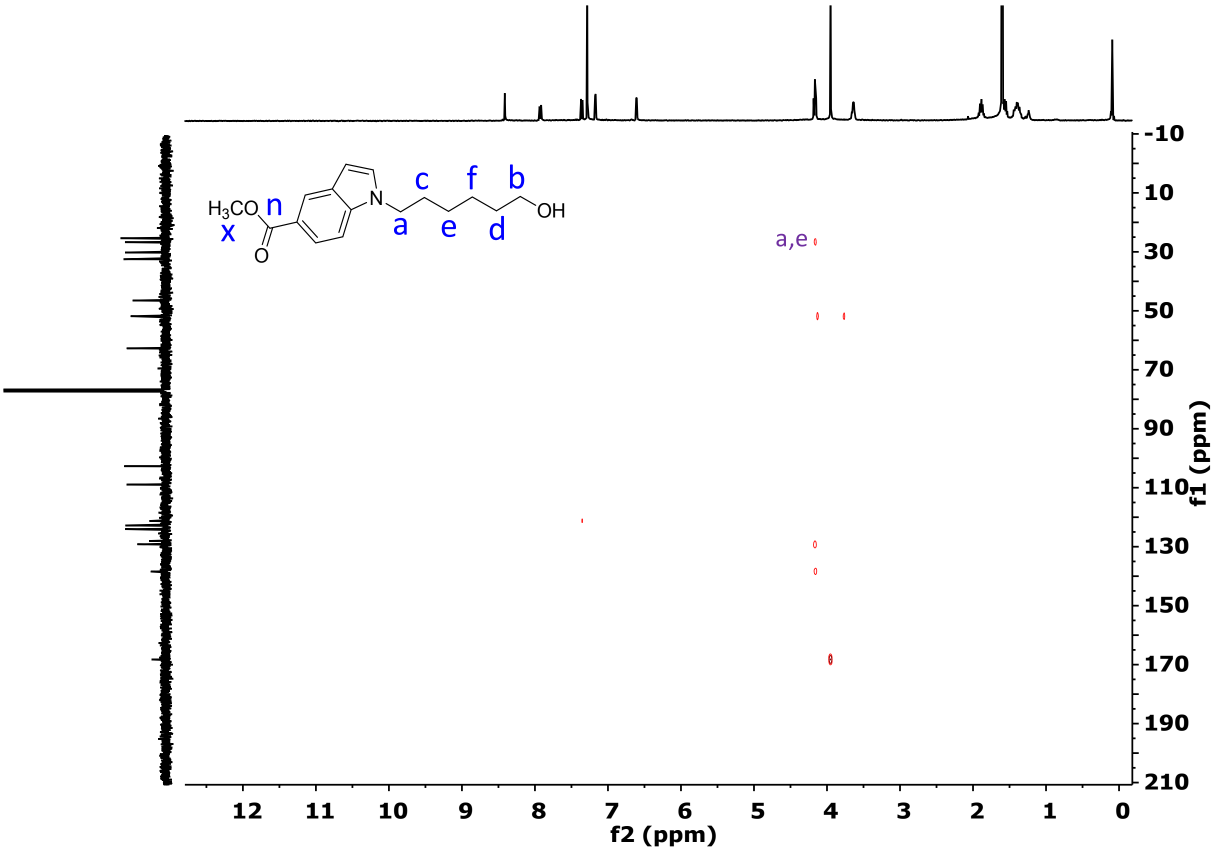

Figure S19 HMBC NMR spectrum of monomer **3d** in $CDCl_3$.

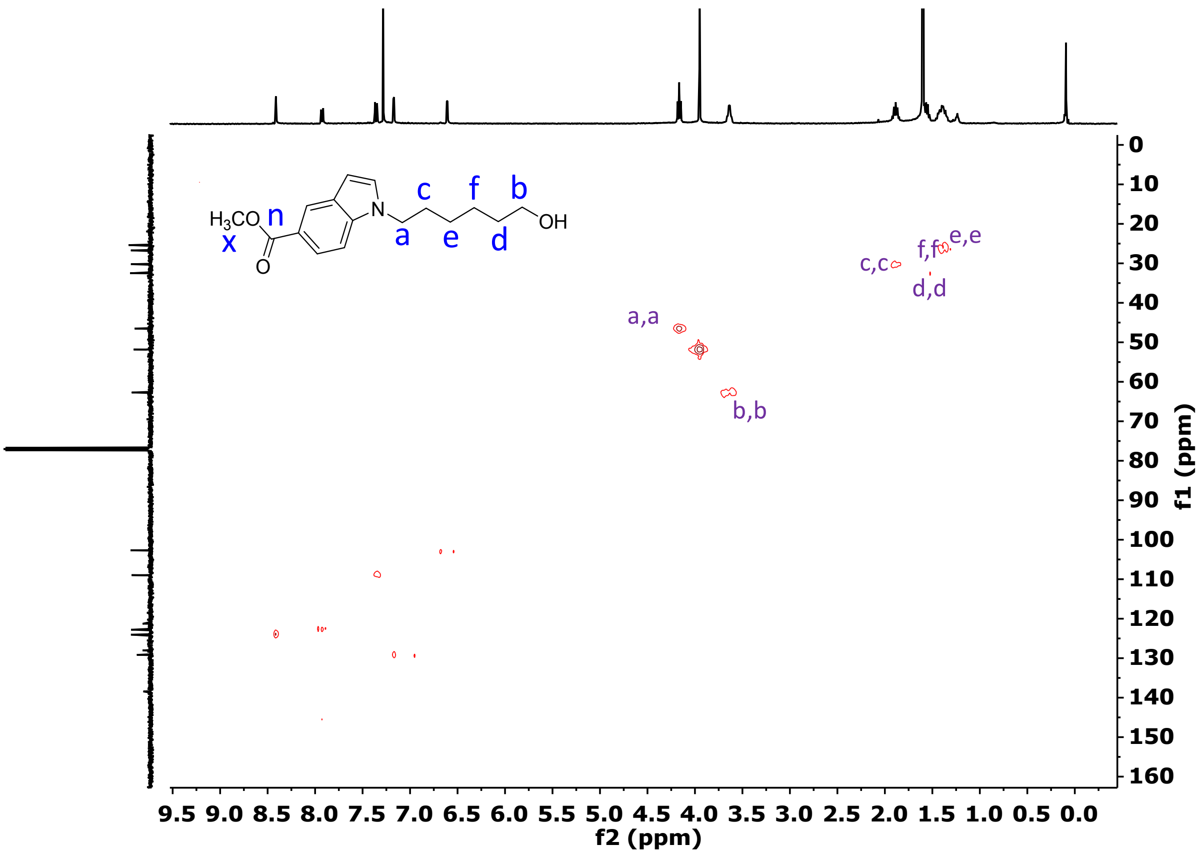

Figure S20 HMQC NMR spectrum of monomer **3d** in $CDCl_3$.

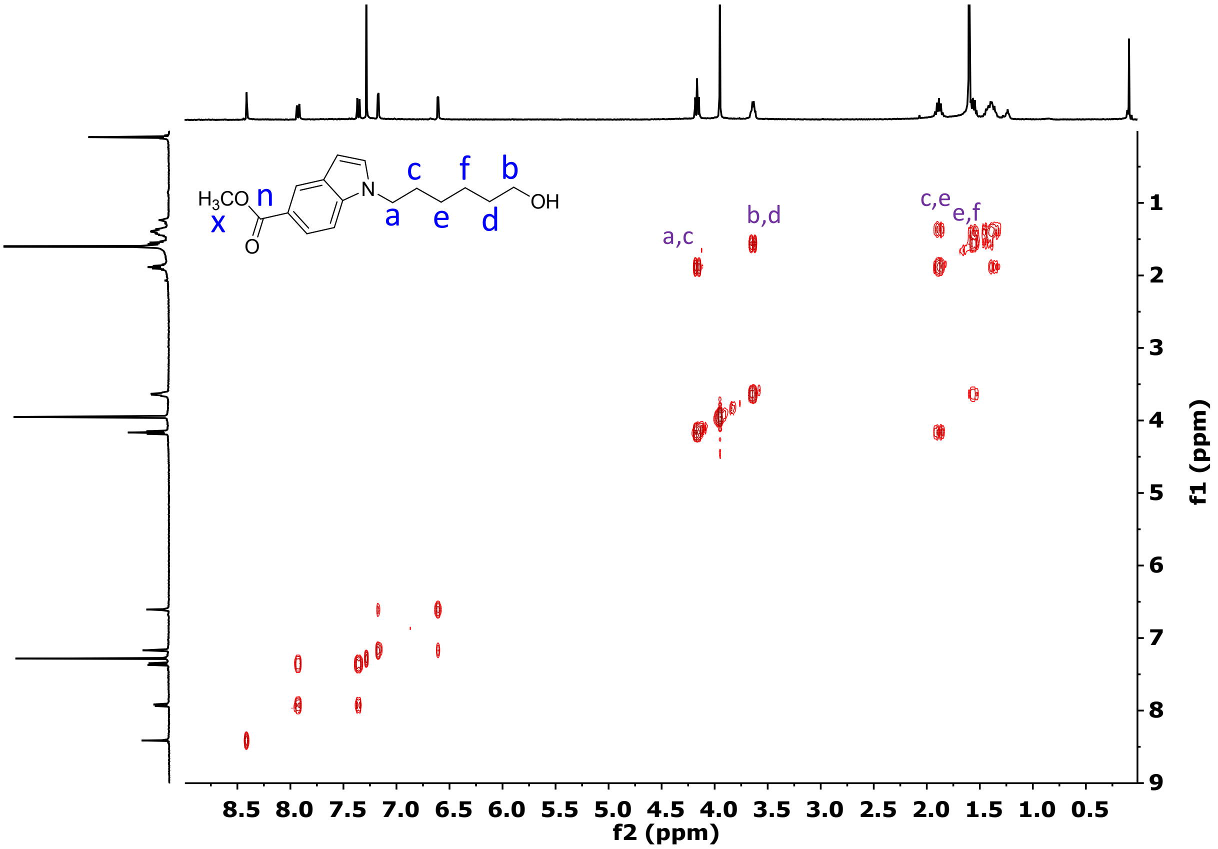

Figure S21 COSY NMR spectrum of monomer **3d** in $CDCl_3$.

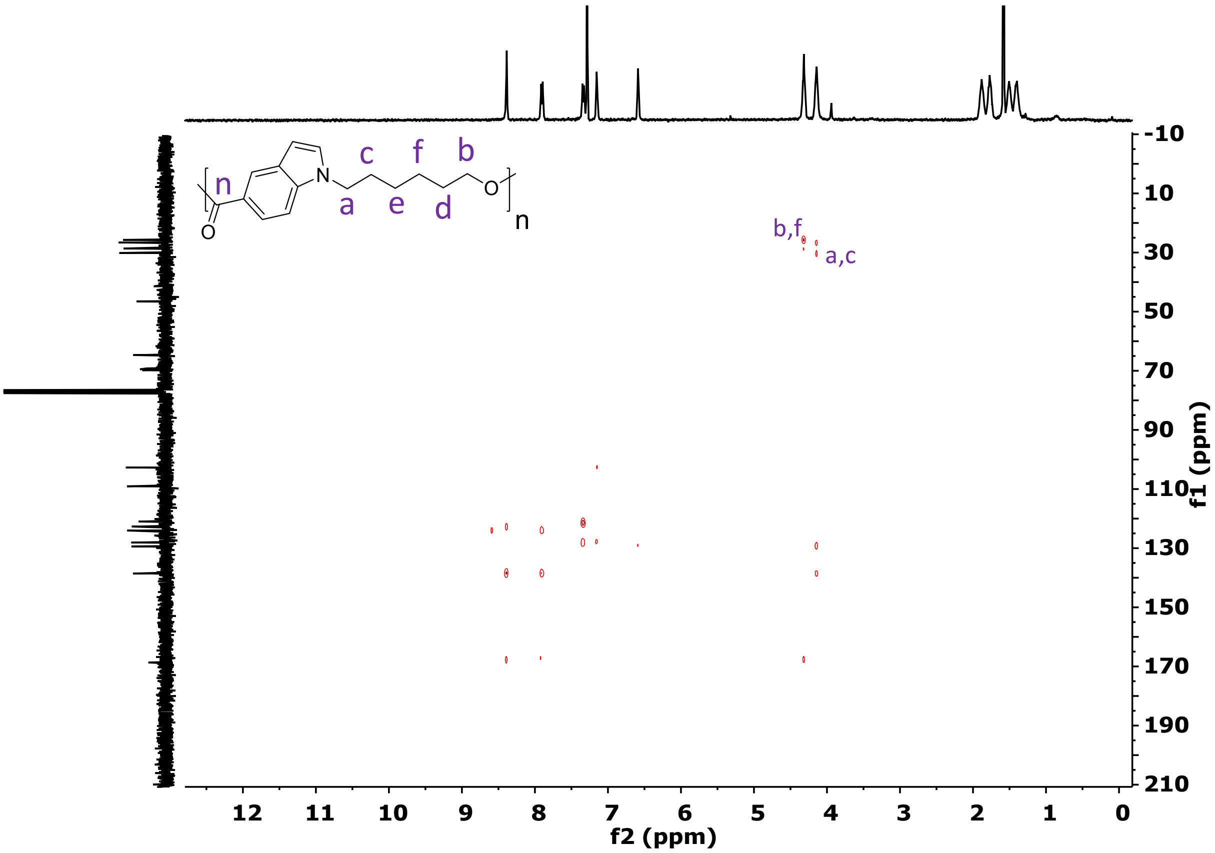

Figure S22 HMBC NMR spectrum of polymer **P3d** in $CDCl_3$.

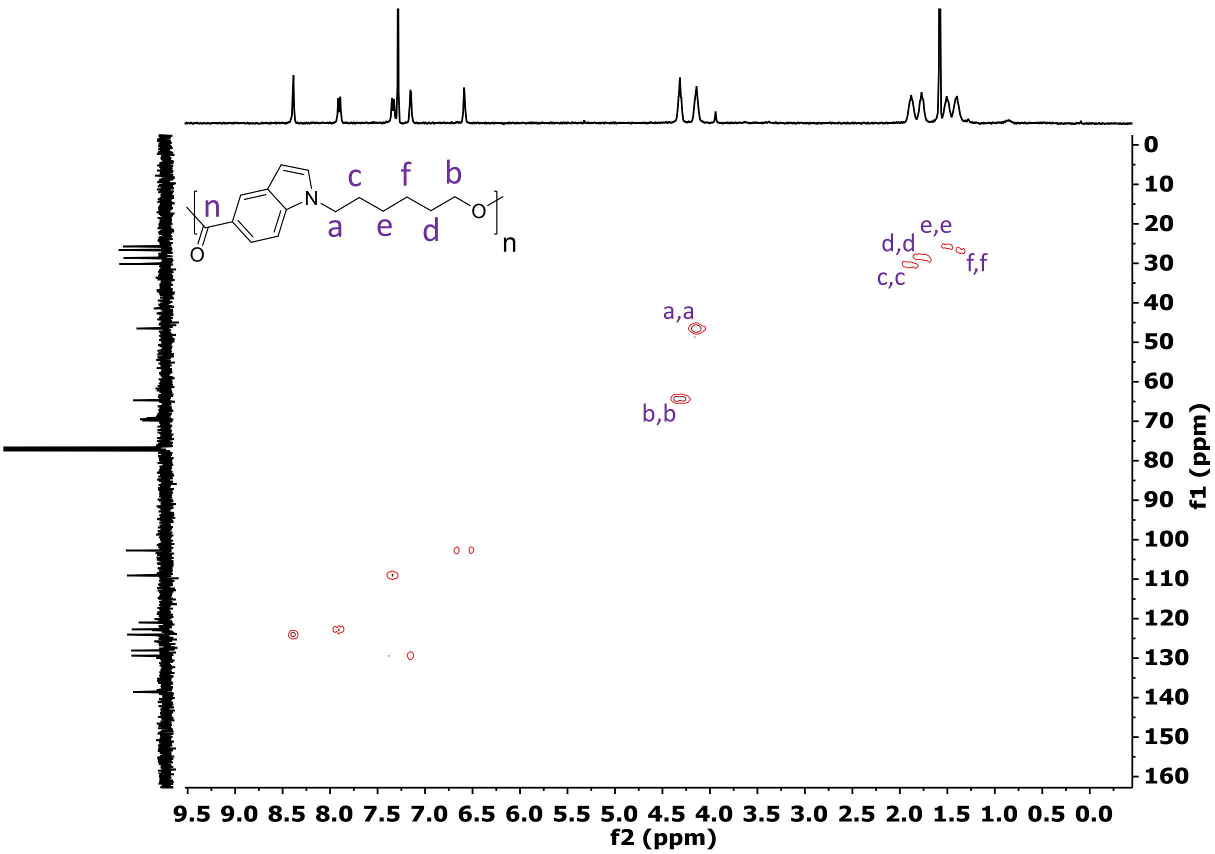

Figure S23 HMQC NMR spectrum of polymer **P3d** in $CDCl_3$.

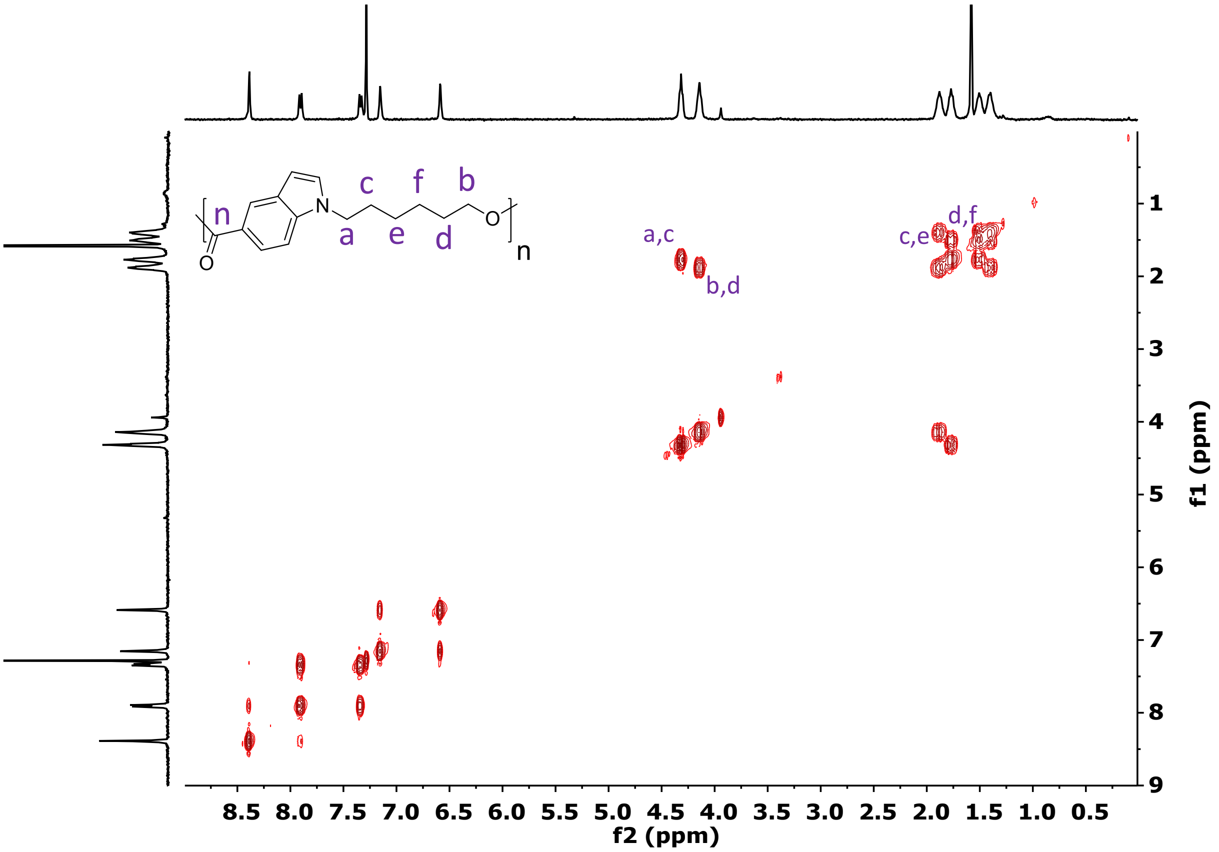

Figure S24 COSY NMR spectrum of polymer **P3d** in $CDCl_3$.

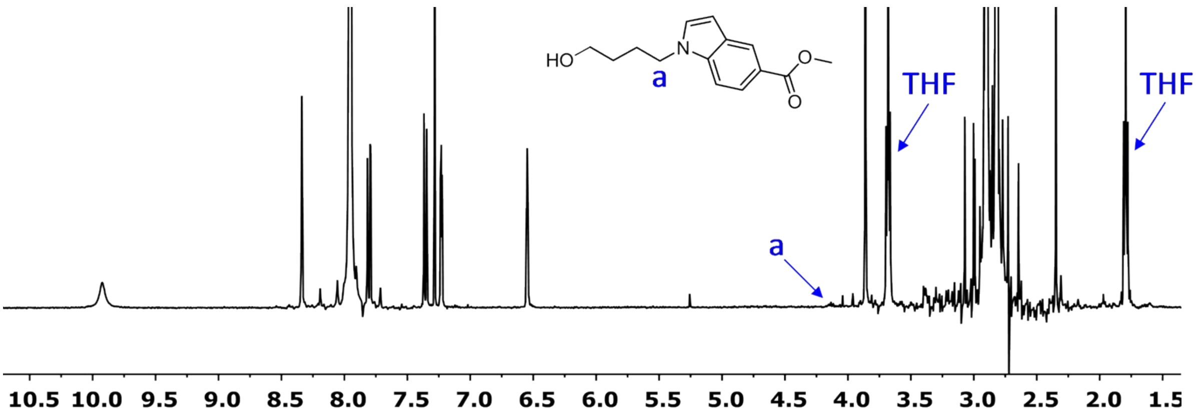

Figure S25 ^{1}H NMR spectrum of the crude reaction mixture of **3b** synthesis after 24 h.

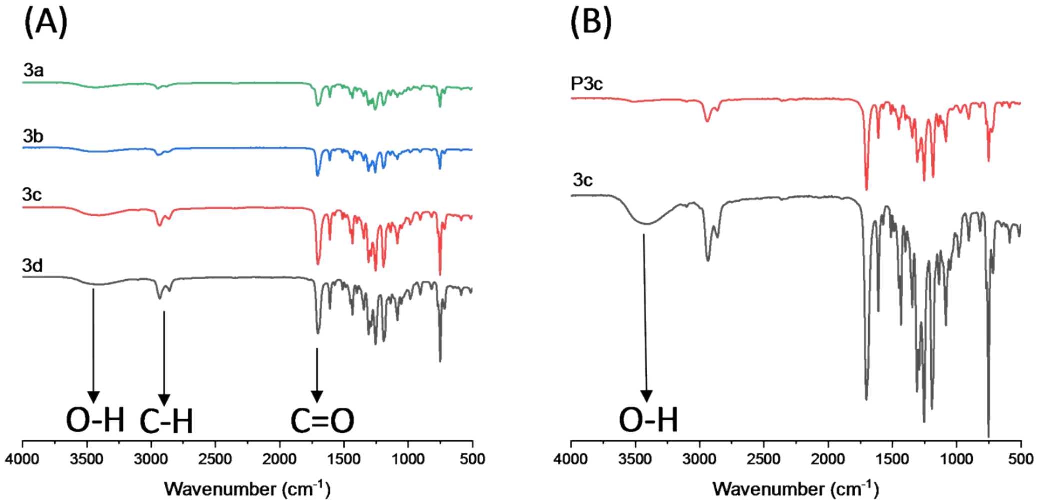

Figure S26 FT-IR spectra of (A) monomers **3a-d** (A), and (B) monomer **3c** and polymer **P3c**.

P4a (m=1)
P4b (m=2)
P4c (m=3)
P4d (m=4)

P5a (m=1)
P5b (m=2)
P5c (m=3)
P5d (m=4)

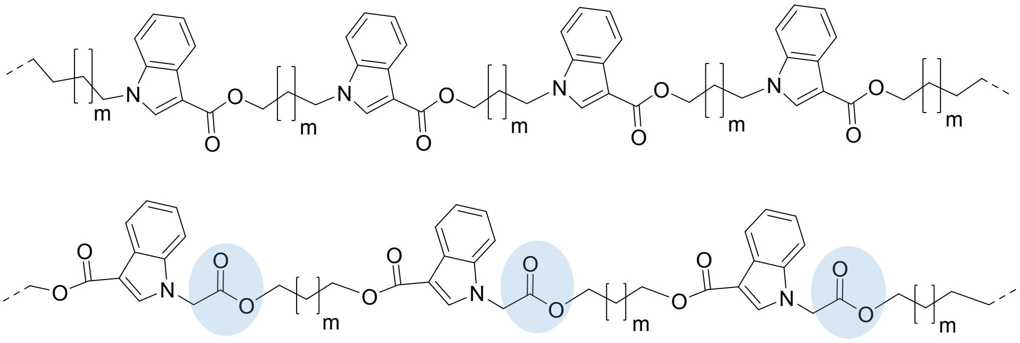

Figure S27 Chemical structures of previously reported polyesters with 1,3-disubstitution patterns. **P4a-d** contain only aromatic-aliphatic esters, while **P5a-d** contain 50% aromatic-aliphatic esters and 50% labile aliphatic-aliphatic esters (shown in blue color).[1,2]

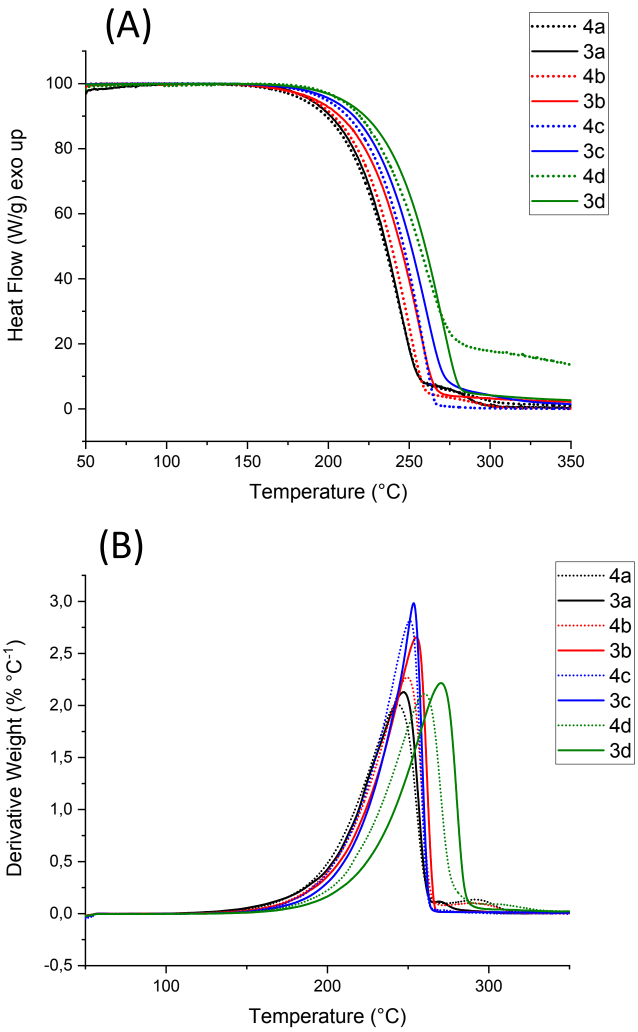

Figure S28 Comparison of the TGA (A) weight loss curves and (B) derivative curves of 1,5-disubstituted monomers **3a-d** (solid curves) and 1,3-disubstituted monomers **4a-d** (dotted curves) reported before.[2]

Table S1 T_d^{95} (temperature for 5% weight loss) and T_d (peak values in the derivative curves) according to the TGA measurements of monomers **4a-d** and **3a-d** (TGA curves shown in Fig. S28).

	**4a**	**3a**	**4b**	**3b**	**4c**	**3c**	**4d**	**3d**
T_d^{95} (°C)	184	188	188	192	198	202	208	208
T_d (°C)	244	247	249	255	252	254	260	270

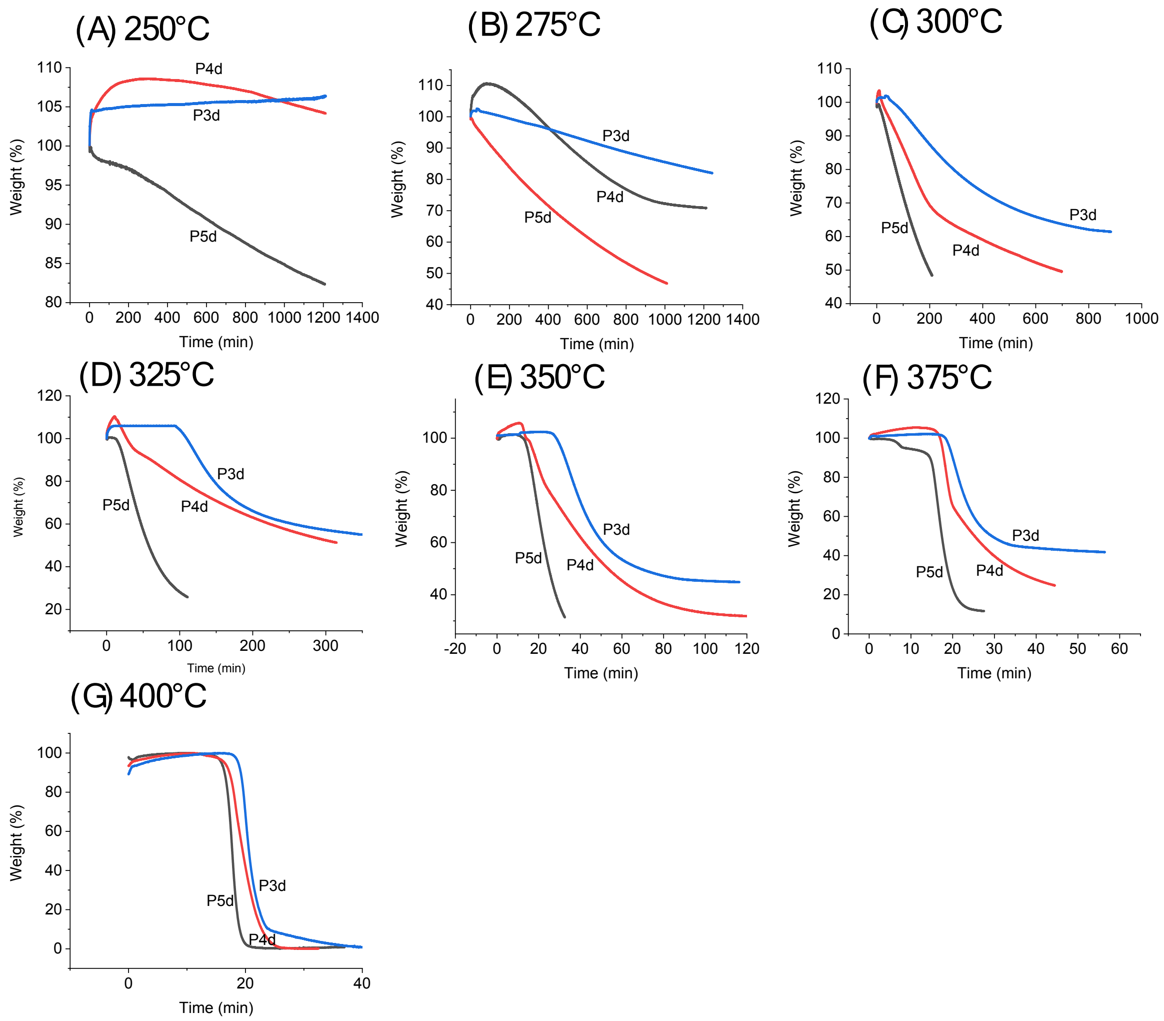

Figure S29 TGA weight-loss curves of polyesters **P3d**, **P4d** and **P5d** (reported earlier) by isothermal heating under nitrogen at the different temperatures, including (A) 250 °C, (B) 275 °C, (C) 300 °C, (D) 325 °C, (E) 350 °C, (F) 375 °C, and (G) 400 °C. The chemical structures of **P4d** and **P5d** are shown in Fig. S27.

Table S2 Time (min) of 10%, 20% and 50% weight loss for **P3d** during isothermal TGA measurements at different temperatures.

Weight loss	250 (°C)	275 (°C)	300 (°C)	325 (°C)	350 (°C)	375 (°C)	400 (°C)

10%	>1200	707	176	115	33	20	19
20%	>1200	>1200	284	136	38	21	20
50%	>1200	>1200	--	--	70	30	21

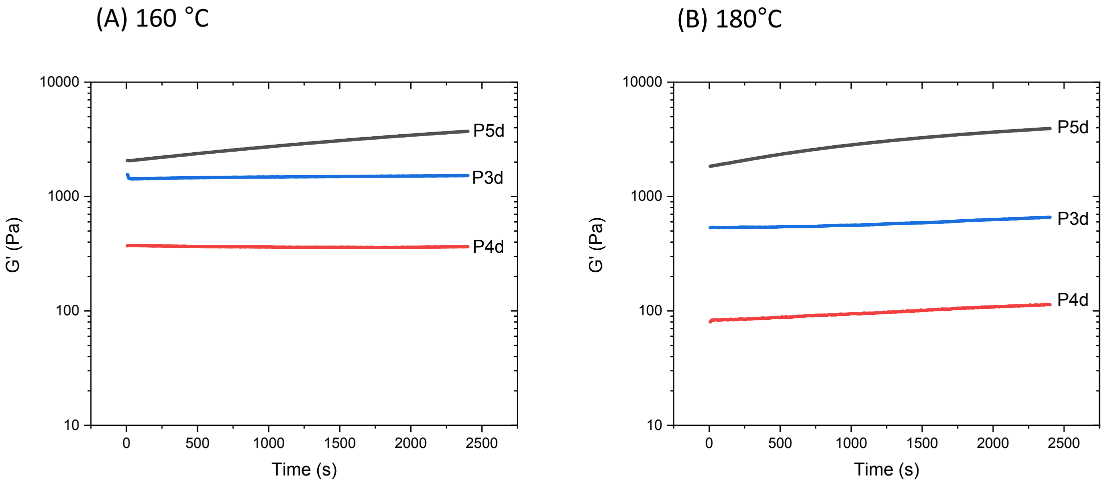

Figure S30. Shear storage modulus of **P3d**, **P4d** and **P5d** measured by rheology at the processing temperatures, 160 °C (A) and 180 °C (B).

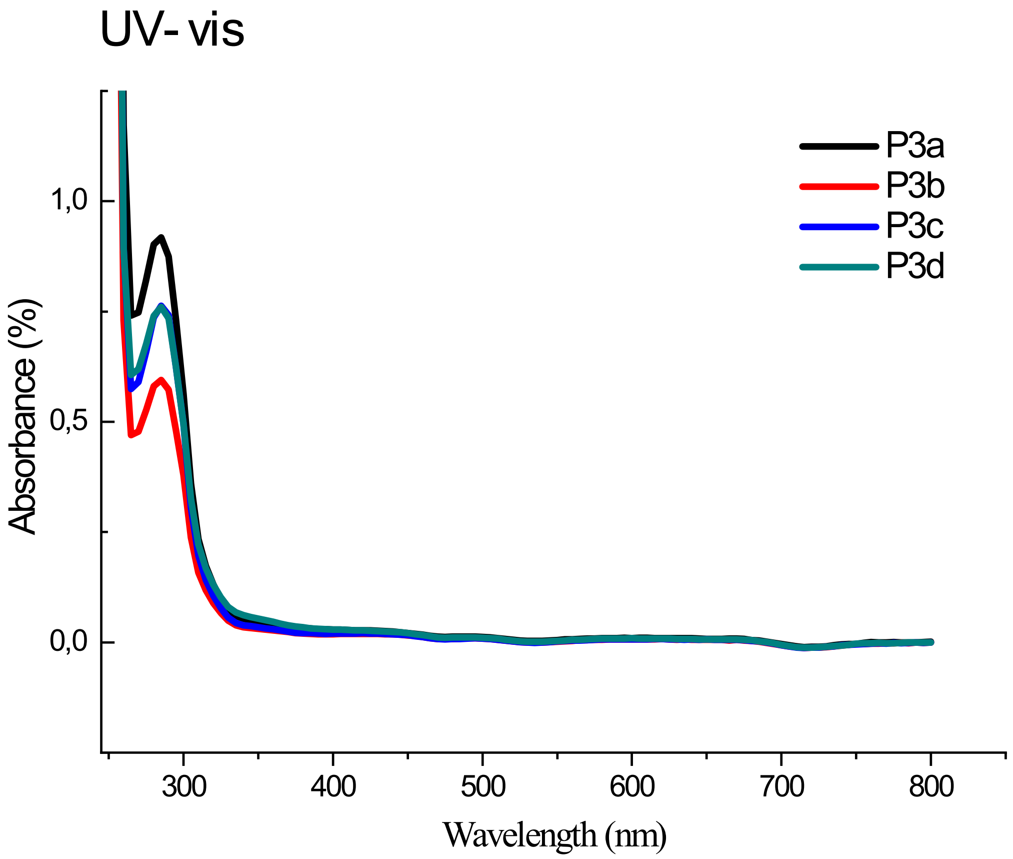

Figure S31 UV-vis spectra of **P3a-d** in chloroform.

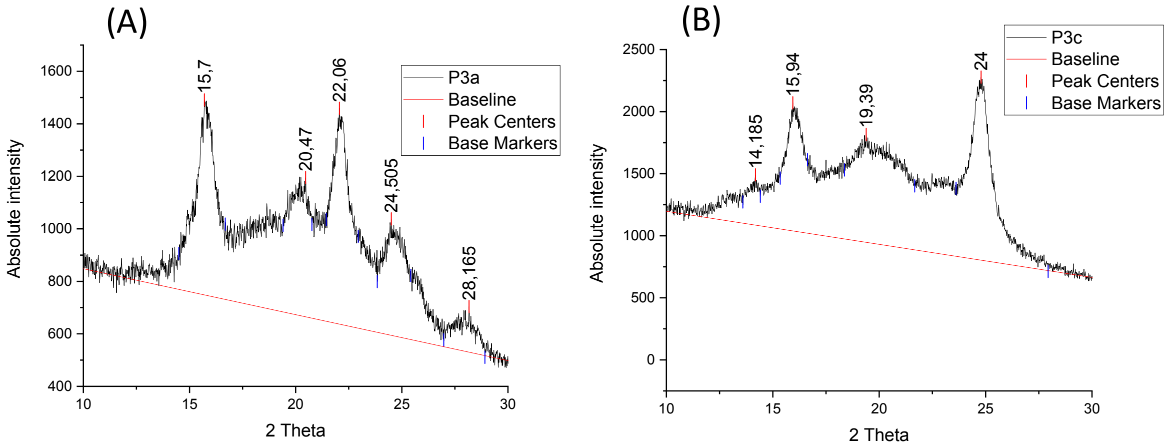

Figure S32 X-ray diffraction patterns of (A) **P3a** and (B) **P3c** solution-casting films.

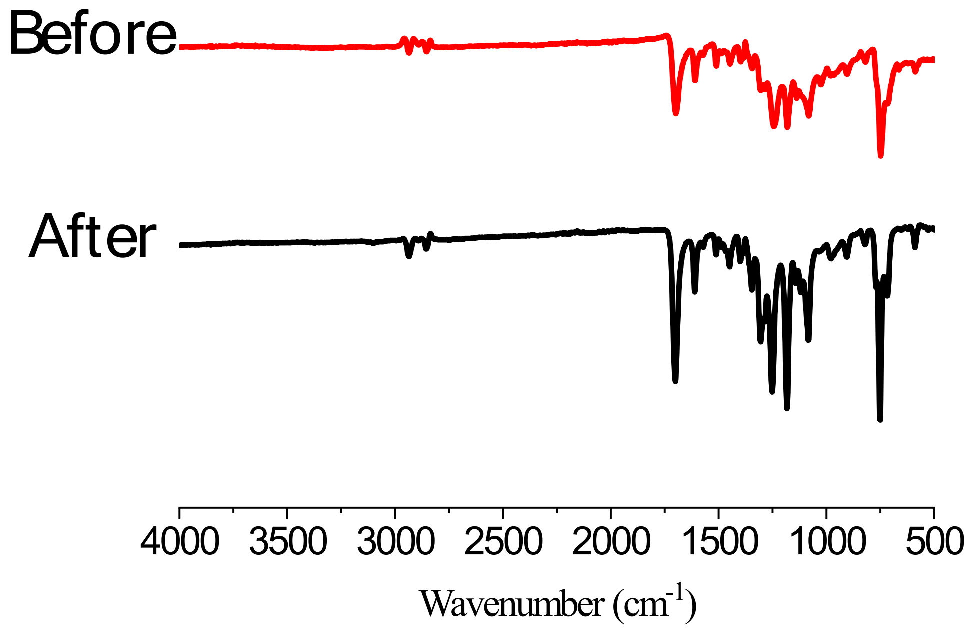

Figure S33 FTIR spectra of **P3d** before and after crosslinking.

References

1 P. Wang, C. R. Arza and B. Zhang, *Polym. Chem.*, 2018, **9**, 4706–4710.

2 P. Wang, J. A. Linares-Pastén and B. Zhang, *Biomacromolecules*, 2020, **21**, 1078–1090.
